# Mesenchymal stromal cells counteract with age-related immune decline and enhance vaccine efficacy by modulating endogenous splenic marginal reticular cells in elderly models

**DOI:** 10.1038/s41423-025-01381-9

**Published:** 2026-01-09

**Authors:** Jialing Liu, Zhishan Li, Qiong Ke, Qiuli Liu, Yueming Sun, Rong Yan, Huolin Ye, Yuxi Zhang, Jie Ren, Hong Chen, Gang Li, Tao Wang, Xubo Li, Yuzhe Wang, Yuan Qiu, Xiaoran Zhang, Zhenxia Yao, Rui Fang, Jianqi Feng, Lili Chen, Weiqiang Li, Xiaoyong Chen, Andy Peng Xiang

**Affiliations:** 1 Hospital of Stomatology, Guanghua School of Stomatology, Sun Yat-sen University, Guangzhou, China; 2 Center for Stem Cell Biology and Tissue Engineering, Key Laboratory for Stem Cells and Tissue Engineering, Ministry of Education, Sun Yat-sen University, Guangzhou, China; 3 National-Local Joint Engineering Research Center for Stem Cells and Regenerative Medicine, Zhongshan School of Medicine, Sun Yat-sen University, Guangzhou, China; 4 Department of Histoembryology and Cell Biology, Zhongshan School of Medicine, Sun Yat-Sen University, Guangzhou, Guangdong China; 5 Biotherapy Center, The Third Affiliated Hospital of Sun Yat-sen University, Guangzhou, China; 6 Department of Medical Ultrasonication, The Third Affiliated Hospital, Sun Yat-sen University, Guangzhou, China; 7 Center for Stem Cells Translational Medicine, Shenzhen Qianhai Shekou Free Trade Zone Hospital, Shenzhen, China; 8 Department of Microsurgery, Orthopedic Trauma and Hand Surgery, The First Affiliated Hospital, Sun Yat-sen University, Guangzhou, China

**Keywords:** Marginal reticular cells (MRCs), Mesenchymal stromal cells (MSCs), Vascular endothelial growth factor A (VEGFA), Vaccine response, Aging, Spleen, Vaccines

## Abstract

Vaccination is the preferred strategy for preventing infections such as influenza in elderly individuals; however, its efficacy is often suboptimal due in part to age-related declines in immune function. In this study, we discovered that the infusion of mesenchymal stromal cells (MSCs) restored defects in the splenic stromal cell network and lymphocyte architecture in aged mice while also increasing specific antibody levels following vaccine immunization. This significantly protected aging mice from influenza infection. Mechanistically, the delivered MSCs localized in the splenic marginal zones, where they positioned themselves near marginal reticular cells (MRCs) and stimulated MRC proliferation, partially through the action of vascular endothelial growth factor A (VEGFA). This MSC‒MRC interaction orchestrated the reconstruction of the stromal network, thereby restoring lymphocyte homeostasis and germinal center reactions. Importantly, the MSC-mediated enhancement of the vaccine response was further validated in aged cynomolgus monkeys. Collectively, our findings provide new insights into the application of MSCs in addressing age-related immune decline and highlight splenic MRCs as critical therapeutic targets.

The decline in immune function among older individuals significantly increases their susceptibility to infections. Adults aged 65 and older experience markedly higher mortality rates from severe influenza and pneumonia [1–3], with over 60% of hospitalized patients in this age group succumbing to influenza infections. Vaccination is the preferred option for preventing influenza in elderly individuals because antiviral therapy post infection has limited effectiveness in reducing mortality [4, 5]. However, the efficacy and effectiveness of vaccination are notably diminished in older adults [6, 7], which is linked to age-related immunodeficiency characterized by a gradual deterioration in immune function [8]. How to enhance vaccine responses in elderly individuals remains a challenge.

The primary goal of vaccination is to establish long-lasting protective immunity, a process fundamentally driven by the germinal center (GC) reaction occurring in the spleen and other secondary lymphoid organs. However, the GC reaction is significantly diminished during aging, resulting in a decline in the functionality of the immune system [9]. The initiation and maturation of GCs largely depend on B cells and their interactions with regulatory supporting cells within the microenvironment, particularly stromal cells in secondary lymphoid organs [10, 11]. Therefore, impaired vaccine responses during aging may arise from both B-cell-intrinsic and B-cell-extrinsic factors [12]. Interestingly, in vivo adoptive transfer studies have shown that B cells from aged mice do not exhibit intrinsic defects in affinity maturation following immunization [13]. Moreover, the aged splenic microenvironment has been demonstrated to negatively impact the migration and maturation of B cells [9], suggesting that the aged microenvironment may play a predominant role in driving age-related impairments in humoral immunity.

The microenvironment of lymphoid organs is established and maintained by lymphoid stromal cells [14–18]. Aging leads to a reduction in stromal cell populations and structural abnormalities within the spleen [19–21]. Furthermore, the modulation of follicular dendritic cells (FDCs), a specialized subset of stromal cells within the microenvironment, has been shown to significantly reshape the GC response [22, 23]. Depletion of lymphoid stromal cells markedly impairs immune responses and antibody production [24], underscoring their critical role as key modulators of immune responses. Consequently, targeting lymphoid stromal cells may represent a potential therapeutic strategy to increase vaccine efficacy in aging populations. Indeed, TLR4 stimulation during vaccination has been reported to increase MAdCAM-1^+^ lymphoid stromal cell activation and promote the aged GC response [25]. However, this intervention did not lead to a significant increase in antibody titers [25], indicating that the precise mechanisms by which lymphoid stromal cells can be modulated to augment vaccine responses remain to be elucidated.

Mesenchymal stromal cells (MSCs) are multipotent cells capable of self-renewal and differentiation into various mesenchymal lineage tissues. MSCs are ubiquitously distributed across nearly all tissues and play essential roles in the development, maintenance, functionality, and regeneration of most tissues [26, 27]. The minimal immunogenicity of MSCs makes them favorable for allogeneic therapy [28–30]. Upon infusion, MSCs exhibit multitarget therapeutic effects to facilitate tissue repair and maintain homeostasis [31–33]. While MSCs are known for their immunomodulatory functions, their effects are highly context dependent [34, 35]. In a proinflammatory environment such as graft-versus-host disease (GVHD), MSCs adopt a potent immunosuppressive phenotype [28, 36–38]. MSC-based therapies for GVHD have achieved significant milestones and have been approved as potential strategies for immune-mediated disorders [39, 40]. However, in the context of age-related immunosenescence, the primary issue is not excessive inflammation but rather a degenerative and dysregulated immune system [41, 42]. Gustafsson et al. identified thymic stromal cells as critical regulators of the lymphopoietic microenvironment. Postn^+^ stromal cells can be durably engrafted in the atrophic thymus, subsequently recruiting and increasing T-cell neogenesis and thereby enhancing the T-cell response to vaccination. More readily available bone marrow mesenchymal populations expressing Ccl19 have similar effects in aged models [42]. These findings provide a compelling rationale for the use of MSC-based lymphoid tissue regenerative approaches in aged individuals. In this context, MSCs are thought to act as rejuvenating agents by restoring the health of the thymus and lymphoid tissue, thereby reconstituting a functional immune landscape. Our previous studies demonstrated that a substantial proportion of systemically administered MSCs homed to the spleen, promoting spleen enlargement and resistance to infection [43]. On the basis of these findings, we hypothesize that MSC-based therapy might be an ideal strategy for modulating the aged splenic microenvironment and ameliorating adaptive immune responses. In this study, we aimed to investigate whether infused exogenous MSCs can repair stromal cells in aging lymphoid organs and restore decreased immune responsiveness.

## Results

### MSCs restored age-related defects in the splenic stromal cells of aged mice

We first detected alterations in the spleen during aging; in line with previous research [44], the spleen decreased slightly in aged mice (18–21 months) compared with young mice (2–3 months) (Extended Data Fig. 1A–C). The frequency and number of splenic stromal cells were significantly reduced in aged spleens (Extended Data Fig. 1D–F), which was also confirmed by immunofluorescence staining (Extended Data Fig. 1G, H). Additionally, immunofluorescence (Extended Data Fig. 1I) and hematoxylin and eosin (H&E) staining (Extended Data Fig. 1J) revealed a disordered structure and a significant reduction in the size of the white pulp area in the aged spleen. Collectively, these results demonstrate that aging contributes to a decline in splenic stromal cells and compromises the microarchitecture of the spleen.

**Fig. 1:**
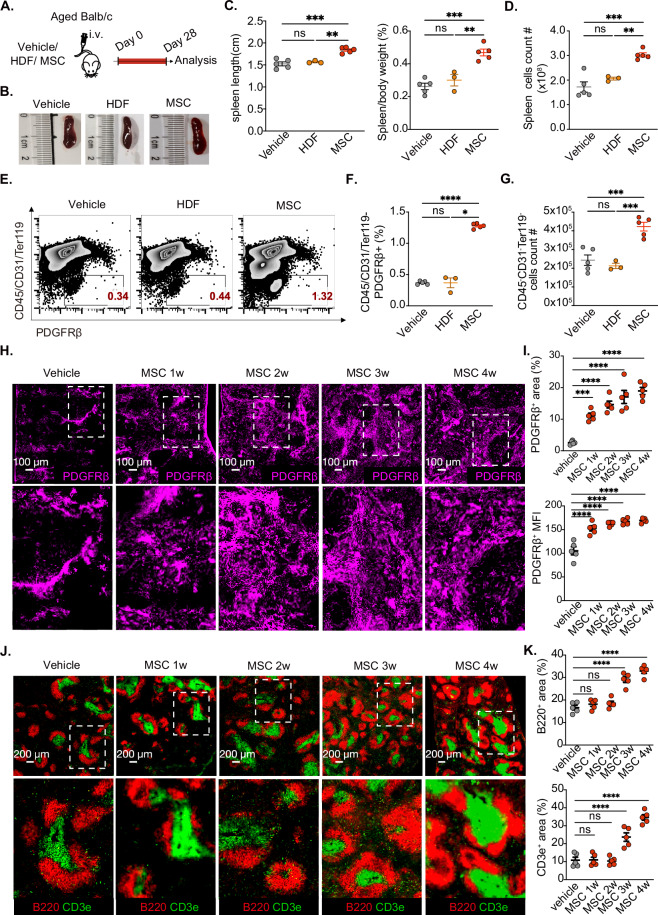
MSCs restored defective splenic stromal architecture in aged mice. **A** Schematic diagram of the experiments; aged (>18-month-old) BALB/C spleen-bearing mice were divided into the following groups: control (Vehicle, *n* = 5), HDF (HDF, *n* = 3), and MSC (MSCs, *n* = 5) groups. **B** Representative spleens from the vehicle, HDF, and MSC groups. **C** Statistical analysis of spleen length and the spleen weight/body weight ratio in the vehicle, HDF and MSC groups. **D** Total splenic cell count in the vehicle, HDF and MSC groups. **E**, **F** Representative dot plots of the splenic CD45^-^CD31^-^Ter119^−^PDGFRβ^+^ stromal cell population and cell counts **G** in the vehicle, HDF, and MSC groups. **H** Representative immunofluorescence staining of splenic stromal cells (PDGFRβ^+^, fuchsia) in the Vehicle and MSC groups (1–4 weeks); scale bars: 100 μm. **I** Statistical analysis of the splenic stromal cell area and mean fluorescence intensity (MFI) in the vehicle and MSC groups (1–4 weeks); five replicate tissues from three fields were quantified per tissue in per group. **J** Representative immunofluorescence staining of splenic T lymphocytes (CD3e^+^, green) and B lymphocytes (B220^+^, red) in the vehicle and MSC groups (1–4 weeks); scale bars: 200 μm. **K** Statistical analysis of the B lymphocyte area and T lymphocyte area in the vehicle and MSC groups (1–4 weeks); five replicate tissues with three fields were quantified per tissue. The data represent the means ± SEMs of three or more independent experiments. Statistical significance was determined via one-way ANOVA with multiple comparisons. *P < 0.05, **P < 0.01, ***P < 0.001, ****P < 0.0001. ns not significant

To functionally validate the unique translational potential of MSCs, human MSCs, rather than nonhuman MSCs, were intravenously administered to aged mice, with human dermal fibroblasts (HDFs) serving as controls because they share a mesodermal origin, similar morphology, in vitro behavior, and partially overlapping surface markers with MSCs [45–49] (Fig. 1A). Twenty-eight days after MSC infusion, the aged spleens were notably larger, with a higher spleen-to-body weight ratio (also referred to as the spleen index in mice) and an increased total number of spleen cells compared with those in the aged control group (Fig. 1B, C). These changes, however, were not observed in the mice that received HDF infusion. Furthermore, MSC infusion significantly increased both the proportion and the number of stromal cells, whereas HDF administration did not induce any notable alterations (Fig. 1E–G). This evidence directly demonstrates that MSC-mediated therapeutic benefits do not stem from nonspecific responses to foreign cells but require orchestrating MSC-specific modulatory machinery.

Immunofluorescence staining further confirmed that MSCs restored the stromal cell network in the aged spleen (Extended Data Fig. 2A–C). Splenic stromal cells began to recover as early as 1 week after the infusion of MSCs and remained well preserved at 4 weeks (Fig. 1H, I). Additionally, the microarchitecture of the spleen recovered, with a significant expansion in the B lymphocyte and T lymphocyte areas following MSC infusion. This effect became observable at 3 weeks and further significantly improved by 4 weeks post-infusion (Fig. 1J, K, Extended Data Fig. 2D–F); at that time, nearly completely infused MSCs were cleared from the spleen (Extended Data Fig. 3A). Collectively, these findings demonstrate that exogenous MSCs can remodel the stromal cell network in the aged spleen, thereby restoring immune cell populations and microarchitectural integrity.

### MSC-mediated spleen rejuvenation promoted germinal center formation and potentiated specific antibody production in aged mice

**Fig. 2:**
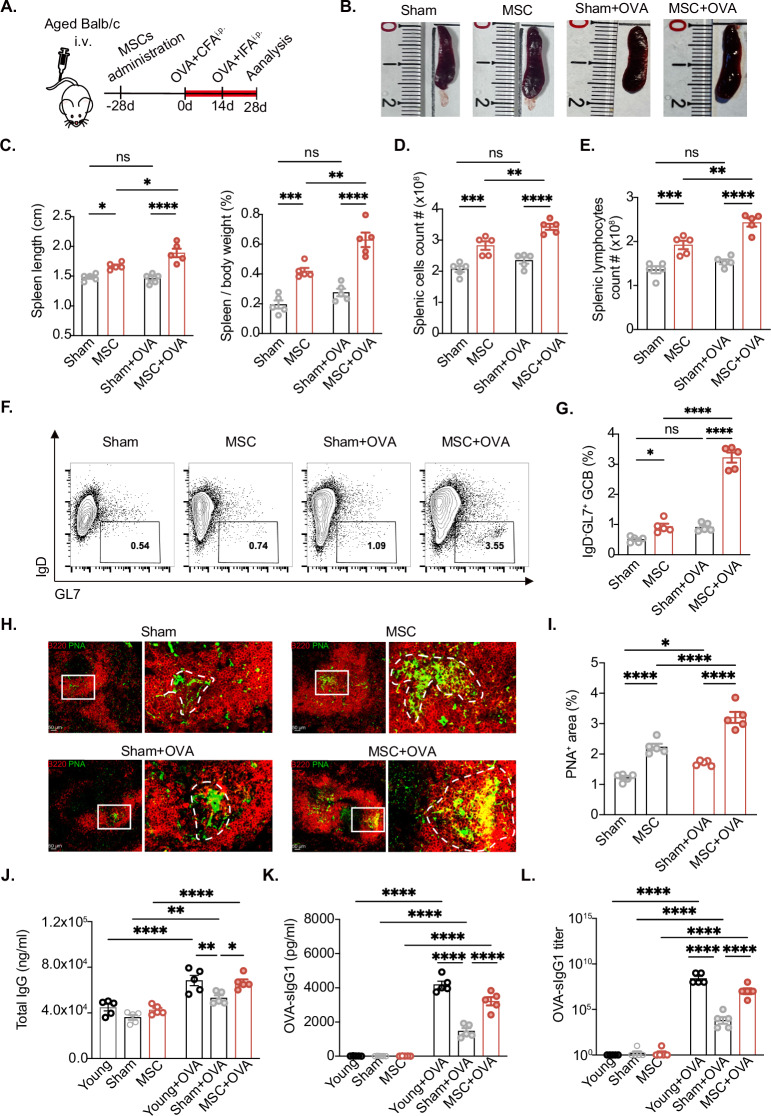
MSCs promote splenic germinal center formation and increase specific antibody levels in aged mice. **A** Schematic diagram of the experiments and ovalbumin (OVA) immunization schedule for aged BALB/C mice. **B** Representative spleen samples from aged BALB/c mice in the Sham, MSC, Sham + OVA and MSC + OVA groups. **C** Statistical analysis of spleen length and the spleen weight/body weight ratio in the sham, MSC, sham + OVA and MSC + OVA groups; *n* = 5 mice per group. **D** Total splenic cell count in the Sham, MSC, Sham + OVA and MSC + OVA groups, *n* = 5 mice per group. **E** Total splenic lymphocyte count in the Sham, MSC, Sham + OVA, and MSC + OVA groups, *n* = 5 mice per group. **F**, **G** Flow cytometry analysis of splenic germinal center B-cell populations (B220^+^IgD^−^GL7^+^) in the Sham, MSC, Sham + OVA, and MSC + OVA groups; *n* = 5 mice per group. **H** Representative immunofluorescence images of B lymphocytes (B220^+^, red) and germinal centers (PNA^+^, green) in the Sham, MSC, Sham + OVA, and MSC + OVA groups; scale bars: 50 μm. **I** Statistical analysis of the splenic germinal center (PNA^+^) area in the Sham, MSC, Sham + OVA, and MSC + OVA groups; *n* = 5 mice per group. **J** Concentrations of total serum IgG1 antibodies after 28 days of immunization in the young (2 months), sham, MSC, sham + OVA, and MSC + OVA groups; *n* = 5 mice per group. (**K**, **L**) Concentrations and titers of serum soluble ovalbumin-specific IgG1 antibodies after 28 days of immunization in the young (2 months), sham, MSC, sham + OVA and MSC + OVA groups; *n* = 5 mice per group. The data represent the means ± SEMs of three or more independent experiments. Statistical significance was determined via one-way ANOVA with multiple comparisons. *P < 0.05, **P < 0.01, ***P < 0.001, ****P < 0.0001. ns not significant

Given that MSCs restored the stromal cells and microarchitecture of the aged spleen, we next explored their potential to mitigate age-related humoral immune decline. As shown in Fig. 2Α, aged mice (18–21 months) were administered MSCs. Twenty-eight days post-treatment, the mice were immunized intraperitoneally with ovalbumin (OVA) in complete Freund’s adjuvant (CFA), followed by a booster immunization with OVA in incomplete Freund’s adjuvant (IFA) 14 days later. While minimal changes were observed in the spleens of the control group following OVA immunization, significant spleen enlargement was evident in the MSC-treated groups, particularly after OVA immunization (Fig. 2B–E). High-affinity antibodies are typically generated through the process of affinity maturation that occurs within germinal centers (GCs) [11, 50], which are often impaired during aging [51]. Thus, we further investigated alterations in GC formation. Intriguingly, the MSC-treated group exhibited a remarkable increase in the CD45^+^B220^+^IgD^−^GL7^+^ germinal center B (GCB) subset (Fig. 2F, G). Additionally, compared with the control, MSC administration resulted in an enlarged peanut agglutinin (PNA)-positive fluorescence area (GC area), providing additional evidence of enhanced GC formation (Fig. 2H, I). Moreover, we found that Tfh cells localized inside GCs to support the GC reaction, further supporting enhanced GC formation following MSC administration following OVA immunization (Extended Data Fig. 4A).

Upon OVA immunization, notable increases in total IgG levels were detected in the serum of young, aged, and MSC-treated mice, confirming immune activation in all groups. Consistent with the findings of GC formation, the aged mice presented markedly lower total IgG levels than their younger counterparts did, and MSC intervention led to a discernible increase in IgG production (Fig. 2J). Importantly, further analysis of OVA-specific IgG levels revealed a more robust antibody response in young mice postimmunization than in aged mice (Fig. 2K, L). Strikingly, MSC treatment significantly elevated both the concentration and titer of OVA-specific IgG in aged mice, highlighting the therapeutic potential of MSCs in enhancing antigen-specific antibody responses in elderly individuals (Fig. 2K, L). Collectively, these findings demonstrate that MSC-mediated rejuvenation of the aged spleen promoted germinal center formation and potentiated specific antibody production through the modulation of splenic stromal cells.

### MSC-mediated vaccine efficacy enhances and protects against influenza infection in aging mice

**Fig. 3:**
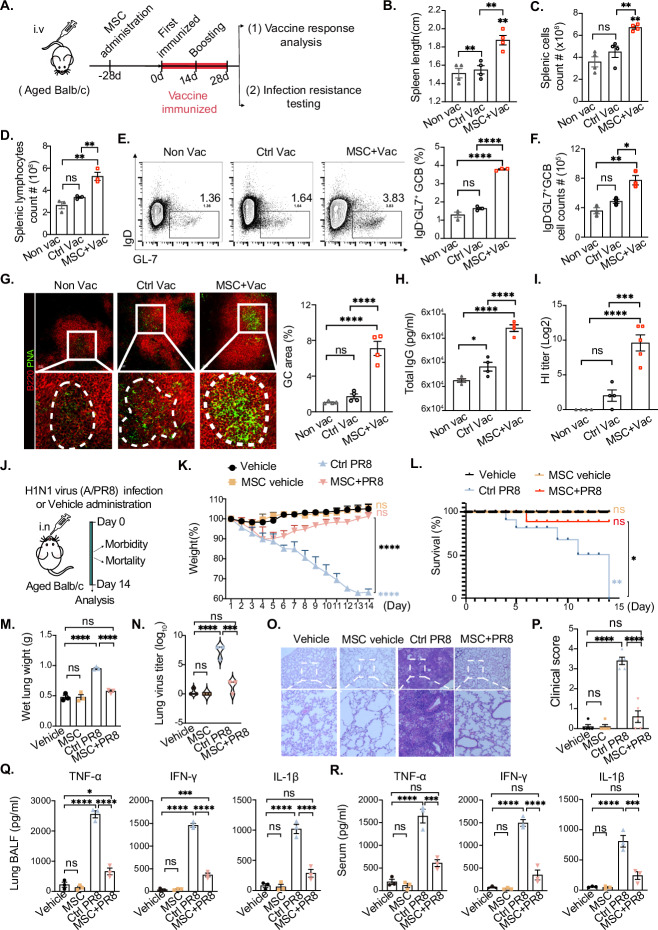
MSCs enhanced vaccine efficacy and protected against influenza infection in aged mice. **A** Quadrivalent inactivated influenza vaccine immunization schedule. **B**, **C** Statistics of the splenic length and splenic cell count in the nonvaccine (Non vac), vaccine-immunized control (Ctrl vac) and vaccine-immunized groups after MSC administration (MSC+Vac), *n* = 4 mice per group. **D** Statistical analysis of splenic lymphocyte counts in the nonvac, Ctrl vac, and MSC+Vac groups; *n* = 3 mice per group. **E** Flow cytometry analysis of splenic germinal center B-cell populations (B220^+^IgD^−^GL7^+^) in the nonvac, Ctrl vac, and MSC+Vac groups; *n* = 3 mice per group. **F** Statistical analysis of splenic germinal center B-cell counts in the nonvac, Ctrl vac, and MSC+Vac groups; *n* = 3 mice per group. **G** Representative immunofluorescence images of B lymphocytes (B220^+^, red) and germinal centers (PNA^+^, green) in the nonvac, Ctrl vac, and MSC+Vac groups; *n* = 4 mice per group. Scale bars: 30 μm. **H** Concentrations and titers of serum soluble IgG antibodies after 28 days of immunization in the nonvac, Ctrl vac, and MSC+Vac groups; *n* = 4 mice per group. **I** Titers of serum vaccine-specific antibodies were measured via a hemagglutination inhibition (HI) test after 28 days of immunization in the nonvac, Ctrl vac, and MSC+Vac groups; *n* = 5 mice per group. **J** H1N1 virus (A/PR8) influenza infection aged mouse model was established after receiving or not receiving quadrivalent split influenza vaccine immunization. **K** Body weight changes in the groups of uninfected aged mice (Vehicle and MSC vehicle) and infected aged mice (Ctrl PR8 and MSC + PR8) over 14 days. **L** Survival analysis of the groups of uninfected aged mice (Vehicle and MSC vehicle) and infected aged mice (Ctrl PR8 and MSC + PR8) over 14 days; *n* = 5 mice per group. **M** Analysis of wet lung weights in mice on day 14 after influenza virus infection in the groups of uninfected aged mouse groups (Vehicle and MSC vehicle) and infected aged mouse groups (Ctrl PR8 and MSC + PR8), *n* = 3 mice per group. **N** Analysis of virus titers in the bronchoalveolar lavage fluid (BALF) of the lungs of uninfected aged mice (Vehicle and MSC vehicle) and infected aged mice (Ctrl PR8 and MSC + PR8) on day 14 after influenza virus infection; *n* = 3 mice per group. **O** Representative immunohistochemical (IHC) hematoxylin‒eosin (HE) staining of lung tissue and clinical score analysis **P** of mice on day 14 after influenza virus infection in the groups of uninfected aged mice (Vehicle and MSC vehicle) and infected aged mice (Ctrl PR8 and MSC + PR8). Scale bars: 250 μm. **Q** Analysis of the levels of inflammatory cytokines (tumor necrosis factor alpha (TNF-α), interferon-γ (IFN-γ) and interleukin-1β (IL-1β)) in the lung BALF of the mice on day 14 after influenza virus infection in the groups of uninfected aged mice (Vehicle and MSC vehicle) and infected aged mice (Ctrl PR8 and MSC + PR8), *n* = 3 mice per group. **R** Analysis of serum inflammatory cytokines (TNF-α, IFN-γ and IL-1β) in mice on day 14 after influenza virus infection in the groups of uninfected aged mice (Vehicle and MSC vehicle) and infected aged mice (Ctrl PR8 and MSC + PR8), *n* = 3 mice per group. The data represent the means ± SEMs of three or more independent experiments. Statistical significance was determined via one-way ANOVA with multiple comparisons. *P < 0.05, **P < 0.01, ***P < 0.001, ****P < 0.0001. ns not significant

Most vaccines for elderly individuals offer limited protection, resulting in increased morbidity and mortality following infection, such as influenza A virus infection [52, 53]. To address this, we evaluated the impact of MSCs administration on vaccine efficacy and protection against influenza infection in aged mice. Four weeks before vaccination, aged mice were administered MSCs (Fig. 3A). To bridge preclinical findings with clinical relevance, the mice were immunized with a quadrivalent influenza vaccine (QIV, the current clinical standard for human vaccination), which received two doses spaced 14 days apart. Consistent with the OVA immunization results, the MSC-treated group exhibited significant spleen enlargement, along with increased splenic lymphocytes and germinal center B cells (Fig. 3B–F), suggesting a more robust vaccine-induced immune response than the control group did. Immunofluorescence staining further confirmed the increased formation of splenic germinal centers in MSC-treated mice (Fig. 3G). Additionally, the MSC-treated group presented a more pronounced increase in total IgG levels postvaccination than did the control group (Fig. 3H). Subsequent hemagglutination inhibition (HI) assays revealed significantly higher serum antibody titers against the influenza vaccine in the MSC-treated group than in the control group (Fig. 3I). These findings indicate that MSC administration significantly enhances the humoral immune response to influenza vaccine vaccination in aged mice.

To systematically evaluate the immune protective potential conferred by MSC-mediated humoral enhancement, we conducted challenge experiments using the influenza strain A/PR8/34 (H1N1) (Fig. 3J), which was selected on the basis of its status as a good laboratory-adapted viral model and the established capacity of QIV-induced antibodies to elicit cross-protective humoral immunity against this particular subtype [54, 55]. Following infection, aged mice exhibited progressive weight loss and succumbed to infection within 2 weeks (Fig. 3K, L). In contrast, MSC-treated mice displayed gradual weight recovery beginning on day 7 post infection, with a 90% survival rate after viral challenge (Fig. 3K, L). Furthermore, while infected control mice presented significant increases in lung wet weight and elevated viral titers in lung tissues, the MSC-treated group presented marked reductions in these indicators (Fig. 3M, N). Histopathological analysis and clinical scores further corroborated the beneficial effects of MSC treatment, revealing reduced lung inflammation and alleviation of clinical symptoms (Fig. 3O, P). Moreover, MSC-treated mice presented significantly lower levels of proinflammatory factors in both lung tissues and systemic circulation following influenza virus infection (Fig. 3Q, R). These findings highlight the potential of MSCs to mitigate age-related humoral immune decline, enhance vaccine responsiveness, and provide effective protection against infections in elderly individuals.

### MSCs rebuild the splenic stromal cell network by promoting the proliferation of splenic marginal reticular cells

Systemic MSCs administration leads to initial pulmonary entrapment, subsequent redistribution to the liver, and significant splenic accumulation [56–58]. Our in vivo two-photon confocal microscopy results also confirmed that MSCs exhibited robust splenic engraftment, minimal hepatic presence, and absence in the kidney/other organs (Extended Data Fig. 3B). Furthermore, we also performed splenectomy experiments to examine the effects of nonspleen targets. As shown in Extended Data Fig. 5, our data demonstrated that MSC-enhanced vaccine responses were completely abrogated postsplenectomy (Extended Data Fig. 5B–D). These findings mechanistically validate that the spleen is a nonredundant lymphoid niche orchestrating MSC-mediated immune potentiation.

**Fig. 4:**
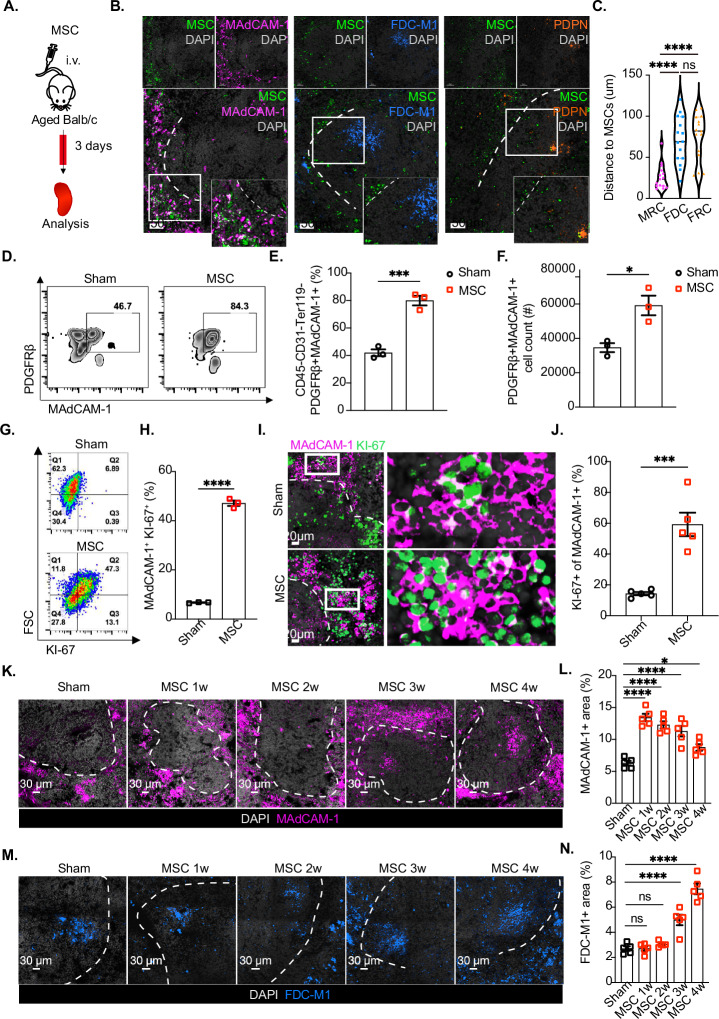
MSCs promoted the proliferation of splenic marginal reticular cells in aged mice. **A** MSCs were administered to aged BALB/c mice (>18 months old) and analyzed 3 days later (**B**–**J**). **B** Representative immunofluorescence staining of MSCs (green), MRCs (MAdCAM-1^+^, fuchsia), FDCs (FDC-M1^+^, blue), FRCs (PDPN^+^, orange), and DAPI (gray) and distance analysis; scale bars: 50 μm. **C** Statistical analysis of the distance between MSCs and each stromal cell population in the spleen. **D** Representative flow cytometry data of splenic MRCs (CD45^−^CD31^-^Ter119^−^ PDGFRβ^+^MAdCAM-1^+^) in the sham and MSC (MSC) groups. **E** Statistical analysis of the proportions of splenic MRCs in the sham and MSC groups; *n* = 3 mice per group. **F** Statistical analysis of splenic MRC cell counts in the sham and MSC groups; *n* = 3 mice per group. **G** Representative flow cytometry analysis of splenic MRC proliferation (CD45^−^CD31^−^Ter119^−^MAdCAM-1^+^KI-67^+^) in the Sham and MSC groups. **H** Statistical analysis of splenic MRC proliferation in the sham and MSC groups; *n* = 3 mice per group. **I** Representative immunofluorescence images of splenic MRCs (MAdCAM-1^+^, fuchsia), Ki-67 (green) and DAPI (gray); scale bars: 20 μm. **J** Statistical analysis of splenic MRC counts in the sham and MSC groups; *n* = 3 mice per group. and statistical analysis of KI-67^+^ MAdCAM-1^+^ cells in the sham and MSC groups, *n* = 5 mice per group. **k** Representative immunofluorescence staining of splenic MRCs (MAdCAM-1^+^, fuchsia) and DAPI (gray) in the spleens of aged mice after vehicle (sham) or MSC administration for 1, 2, 3, and 4 weeks; scale bars: 30 μm. **L** Statistical analysis of the splenic MRC area (MAdCAM-1^+^) in the spleens of aged mice after vehicle (sham) or MSC administration for 1, 2, 3, and 4 weeks; *n* = 5 mice per group. **M** Representative images of FDC (FDC-M1^+^, fuchsia) and DAPI (gray) immunofluorescence staining in the spleens of aged mice after vehicle (sham) or MSC administration for 1, 2, 3, and 4 weeks; scale bars: 30 μm. **N** Statistical analysis of the splenic FDC area (FDC-M1^+^) in the spleens of aged mice after vehicle (sham) or MSC administration for 1, 2, 3, and 4 weeks; *n* = 5 mice per group. The data represent the means ± SEMs of three or more independent experiments. In (**E**, **F**, **H**, **J**), statistical significance was determined via a two-tailed unpaired *t*-test. In **C**, **L**, **N**, statistical significance was determined via one-way ANOVA with a multiple comparison test. *P < 0.05, **P < 0.01, ***P < 0.001, ****P < 0.0001. ns not significant

We then explored the mechanisms underlying MSC-mediated remodeling of the aging spleen. Using NDG immunodeficient mice, which are devoid of T cells, B cells, and natural killer (NK) cells, we found that MSCs effectively restored the stromal architecture, suggesting that this process may occur independently of immune cell participation (Extended Data Fig. 6A). To further investigate this, we employed *Pdgfrβ*-Cre mice, in which splenic stromal cells underwent targeted ablation via diphtheria toxin administration following delivery of a Cre-dependent adeno-associated viral construct (PAV-CAG-DIO-DTR-P2A-mCherry). This conditional system selectively depleted almost all splenic stromal cells without affecting PDGFRβ^+^ cells in other organs, even the liver, which is closely related to the spleen (Extended Data Fig. 7). Intriguingly, MSCs failed to reconstruct the T and B-cell microenvironments in these mice (Extended Data Fig. 6B), which consequently led to the inability to increase OVA-induced antibody levels following MSCs administration (Extended Data Fig. 8A–H). Moreover, during the early phase after MSC injection (Fig. 4A), we detected a notable increase in the proliferation (Ki-67 expression) of spleen cells (Extended Data Fig. 9A–C), particularly within the CD45^-^ subpopulation (Extended Data Fig. 9C, D). This observation was further corroborated by the finding that remodeling of splenic stromal cells preceded changes in T and B cells (Fig. 1J–K). Taken together, these results suggest that splenic stromal cells may serve as the prerequisite cellular target population for MSC-mediated intervention.

There are three principal groups of stromal cells in the spleen, namely, marginal reticular cells (MRCs), follicular dendritic cells (FDCs), and fibroblastic reticular cells (FRCs) [59]. We found that many MSCs were distributed within the spleen and predominantly localized to the marginal zone underpinned by MRCs, which express the adhesion molecule MAdCAM-1 [60] (Fig. 4B, C). Notably, the proportion and absolute number of MRCs (identified as CD45^-^CD31^-^Ter119^-^PDGFRβ^+^MAdCAM-1^+^) significantly increased after MSC infusion (Fig. 4D–F), suggesting that MAdCAM-1^+^ MRCs may represent the primary cellular target of MSCs. This finding was further supported by the MSC-mediated cell proliferation results, which revealed an increase in Ki-67^+^ cells within MAdCAM-1^+^ MRCs after MSCs administration (Fig. 4G–J, Extended Data Fig. 10A) but not in FDCs or FRCs (Extended Data Fig. 11A, B). In addition, quantitative spatial analysis further demonstrated that Ki-67⁺ MRCs reside significantly closer to MSCs (*p* < 0.01) than Ki-67⁻ MRCs do, with an approximately 40% reduction in distance (Extended Data Fig. 10B). Extending the observation period, we analyzed aged spleens at 1–4 weeks postMSC administration. We found that the number of MAdCAM-1^+^ MRCs significantly increased as early as the first week and was well maintained throughout 2–4 weeks (Fig. 4K, L). Intriguingly, MAdCAM-1^+^ cells were also detected in the white pulp area at 3–4 weeks after MSC administration, which coincided with a gradual increase in FDC-M1^+^ FDCs (Fig. 4M, N).

Moreover, using *Madcam1*-Cre/ERT2 × *ROSA26-iDTR* mice, we specifically depleted MAdCAM-1^+^ cells through diphtheria toxin administration. This approach selectively targeted splenic marginal reticular cells (MRCs) while maintaining follicular dendritic cell (FDC) populations and overall lymphoid architecture (Extended Data Fig. 12A–D, and 13A–C). Crucially, MRC ablation completely abrogated the MSC-mediated enhancement of both germinal center responses (Extended Data Fig. 13D) and antigen-specific antibody production following OVA immunization (Extended Data Fig. 13E). These findings provide definitive evidence that MAdCAM-1^+^ MRCs serve as the primary cellular mediators of MSC immunomodulation in the spleen. On the basis of these findings, we propose that MSCs promote the proliferation of MRCs in aged spleens, subsequently increasing the population of FDCs, which are critical for GC promotion and vaccine response.

### MSCs promote MRC proliferation and regenerate the stromal cell network partially through VEGFA

**Fig. 5:**
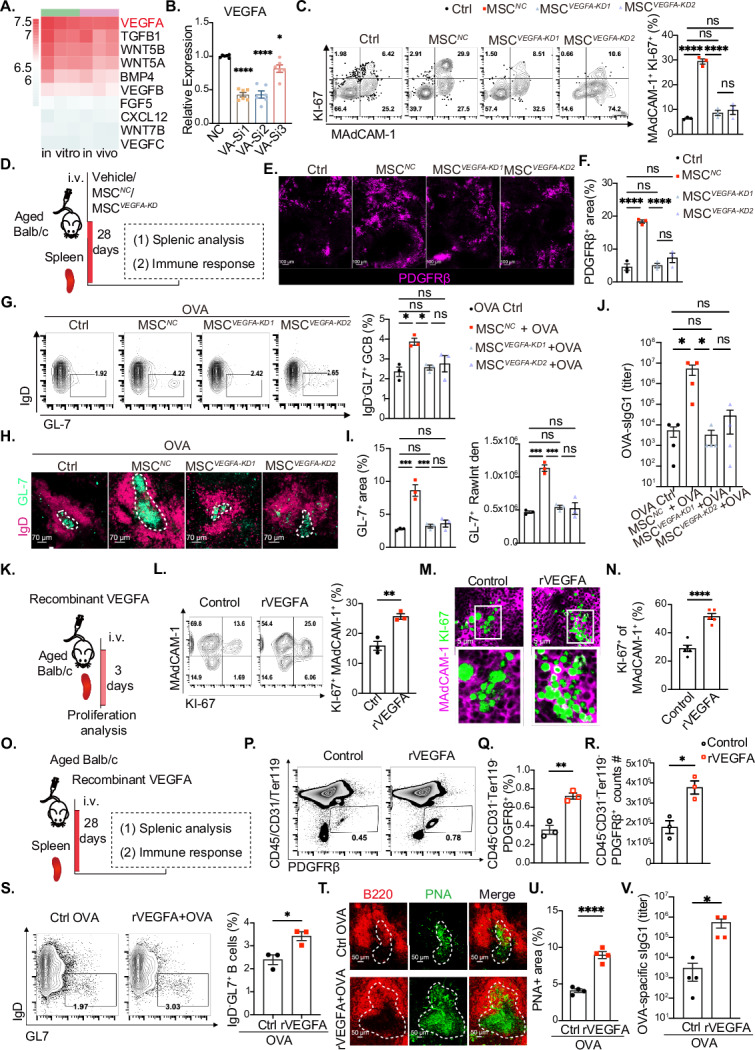
VEGFA is a potential functional molecule by which MSCs promote MRC regeneration. **A** The top 10 ligands were screened and ranked via bulk RNA sequencing data from MSCs in vitro and those distributed in aged spleens. **B** Relative VEGFA mRNA expression was detected through RT‒PCR validation in the MSC^*NC*,^ MSC^*VEGFA-KD1*^, MSC^*VEGFA-KD2*^, and MSC^*VEGFA-KD3*^ groups (*n* = 5). **C** Flow cytometry analysis of splenic MRC proliferation (CD45^-^CD31^-^Ter119^-^MAdCAM-1^+^KI-67^+^) in the Ctrl, MSC^*NC*,^ MSC^*VEGFA-KD1*^, and MSC^*VEGFA-KD2*^ groups; *n* = 3 mice per group. **D** Vehicle (Ctrl), nonsense control MSC (MSC^*NC*^) or VEGFA-knockdown MSC (MSC^*VEGFA-KD*^) were administered to aged BALB/C mice (>18 months old), which were analyzed 28 days later (**E**, **F**) or then immunized with OVA (**G**‒**J**). **E** Representative immunofluorescence staining of splenic stromal cells (PDGFRβ^+^, fuchsia) in the control, MSC^*NC*^, MSC^*VEGFA-KD1*^, and MSC^*VEGFA-KD2*^ groups. Scale bars: 100 μm. **F** Statistical analysis of the proportion of splenic stromal cells in the Ctrl, MSC^*NC*^, MSC^*VEGFA-KD1*^, and MSC^*VEGFA-KD2*^ groups; *n* = 3 mice per group. **G** Flow cytometry analysis of splenic germinal center B-cell populations (B220^+^IgD^-^GL7^+^) 28 days after OVA immunization in the control (OVA Ctrl), MSC^*NC*^ + OVA, MSC^*VEGFA-KD1*^ + OVA, and MSC^*VEGFA-KD2*^ + OVA groups. **H** Representative immunofluorescence staining of follicular B lymphocytes (IgD^+^, fuchsia), germinal centers (GL-7^+^, green), and DAPI (blue) in the OVA Ctrl, MSC^*NC*^ + OVA, MSC^*VEGFA-KD1*^ + OVA, and MSC^*VEGFA-KD*^ groups; scale bars: 70 μm. **I** Statistical analysis of the splenic GC area (GL-7^+^) and GL-7^+^ RawIntden population in the OVA Ctrl, MSC^*NC*^ + OVA, MSC^*VEGFA-KD1*^ + OVA and MSC^*VEGFA-KD2*^ + OVA groups; *n* = 3 mice per group. **J** Titers of serum soluble ovalbumin-specific IgG1 antibodies after 28 days of immunization in the OVA Ctrl, MSC^*NC*^ + OVA, MSC^*VEGFA-KD1*^ + OVA and MSC^*VEGFA-KD2*^ + OVA groups; *n* = 4 mice per group. **K** Recombinant VEGFA was administered to aged BALB/C mice (>18 months old), and the results were analyzed 3 days later (L‒N). **L** Flow cytometry analysis of splenic MRC proliferation (CD45^-^CD31^-^Ter119^-^MAdCAM-1^+^KI-67^+^) in the vehicle (control) or recombinant VEGFA delivery (rVEGFA) groups after 3 days; *n* = 3 mice per group. (**M**) Representative immunofluorescence images of splenic MRCs (MAdCAM-1^+^, fuchsia), Ki-67 (green), and DAPI (gray) in the control and rVEGFA groups; scale bars: 5 μm. **N** Statistical analysis of splenic MRC proliferation in the control and rVEGFA groups; *n* = 5 mice per group. **O** Recombinant VEGFA was administered to aged BALB/C mice (>18 months old), and the results were analyzed 28 days later (**P**–**R**) or after ovalbumin immunization (**S**–**V**). **P** Representative flow cytometry of splenic stromal cells (CD45^-^CD31^-^Ter119^-^PDGFRβ^+^) in the control and rVEGFA groups. **O** Statistical analysis of the proportion of splenic stromal cells in the control and rVEGFA groups; *n* = 3 mice per group. **R** Statistical analysis of splenic stromal cell counts in the control and rVEGFA groups; *n* = 3 mice per group. **S** Flow cytometry analysis of splenic germinal center B-cell populations (B220^+^IgD^−^GL7^+^) in the control (OVA Ctrl) and recombinant VEGFA groups 28 days before OVA immunization (rVEGFA +OVA); *n* = 3 mice per group. **T** Representative immunofluorescence staining of B lymphocytes (B220^+^, red) and germinal centers (PNA^+^, green) in the OVA Ctrl and rVEGFA +OVA groups; scale bars: 50 μm. **U** Statistical analysis of the splenic GC area (PNA^+^) in the OVA Ctrl and rVEGFA +OVA groups; *n* = 4 per group. **V** Titers of serum soluble ovalbumin-specific IgG1 antibodies after 28 days of OVA immunization in the groups of no immunization (Control), sham control (OVA Ctrl), and recombinant VEGFA administration 28 days before OVA immunization (rVEGFA +OVA), *n* = 4 mice per group. The data represent the means ± SEMs of three independent experiments. In (**C**, **F**, **G**, **I**, **J**), statistical significance was determined via one-way ANOVA with a multiple comparison test. In (**L**, **N**, **Q**, **R**, **S**, **U**, **V**), statistical significance was determined via a two-tailed unpaired *t*-test. *P < 0.05, **P < 0.01, ***P < 0.001, ****P < 0.0001. ns not significant

To elucidate the molecular mechanisms underlying the MSC-mediated promotion of MRC proliferation, we performed a comprehensive analysis of potential interactions between spleen MRCs and MSCs via bulk RNA sequencing data. On the basis of ligand‒receptor expression profiles, we initially identified the top 67 receptors associated with cell proliferation, ranked by their expression levels in aged MRCs (GSE171124, Extended Data Fig. 14A). The corresponding ligands were subsequently screened and prioritized according to their expression levels in both in vitro-cultured MSCs and MSCs located in the spleens of aged mice postinfusion (Extended Data Fig. 14B). Among these, VEGFA emerged as the most highly expressed ligand in MSCs (Fig. 5A). VEGFA has been demonstrated to promote healthy aging, extend the lifespan of aging mice [61], and play a pivotal role in tissue repair [62, 63]. We further investigated whether VEGFA mediates the regulation of endogenous splenic stromal cells by MSCs. First, VEGFA expression in MSCs was selectively knocked down via three distinct small interfering RNAs (siRNAs, MSC^*VEGFA-KD*^), with ^*MSCs*^ (nonspecific siRNA controls) serving as the control. A reduction in VEGFA expression was confirmed at both the mRNA and protein levels in the MSC^*VEGFA-KD1*^ and MSC^*VEGFA-KD2*^ groups (Fig. 5B, Extended Data Fig. 15A, B). The therapeutic effects of MSC^*VEGFA-KD1*^, MSC^*VEGFA-KD2*^ and MSC^*NC*^ were subsequently evaluated (Extended Data Fig. 15C). Notably, VEGFA knockdown in MSCs significantly attenuated their capacity to promote MRC proliferation in aged spleens on day 3 (Fig. 5C and Extended Data Fig. 15D). The therapeutic effects of these compounds continued to be assessed (Fig. 5D and Extended Data Fig. 15E). The MSC^*NC*^ group maintained an increase in the spleen weight-to-body weight ratio and spleen cell number, whereas both MSC^*VEGFA-KD*^ groups exhibited diminished effects (Extended Data Fig. 15F). Additionally, VEGFA knockdown in MSCs impaired their ability to restore the splenic stromal network (Fig. 5E, F) and rebuild the splenic B and T-cell architecture (Extended Data Fig. 15G). In the OVA-immunized group, a reduction in the proportion of GC B cells (Fig. 5G) and GC areas was observed in the spleens of the MSC^*VEGFA-KD*^ groups (Fig. 5H, I), accompanied by limited production of OVA-specific IgG1 compared with that in the MSC^*NC*^ group (Fig. 5J).

To further explore the potential of VEGFA in reconstructing the stromal cell network (Fig. 5K), we administered VEGFA to aged mice and observed a significant increase in the Ki-67^+^ population of MRCs compared with that in mice in the vehicle control group on day 3 (Fig. 5L–N). The effects of VEGFA treatment were subsequently assessed (Fig. 5O), revealing a marked expansion of splenic stromal cells in aged mice (Fig. 5P–R). Moreover, VEGFA treatment promoted the formation of GCs after OVA immunization (Fig. 5S–U) and effectively elevated the serum levels of OVA-specific IgG1 in aged mice (Fig. 5V). These results demonstrated that VEGFA plays an important role in promoting MRC proliferation and regenerating the stromal cell network, thereby augmenting immune responses.

In addition, we employed MSC/MRC coculture systems with pharmacological receptor blockade to confirm direct effects on MAdCAM1^+^ cells. By systematically assessing VEGFA signaling through the selective small-molecule antagonists ZM306416 (VEGFR1-specific), Ki8751 (VEGFR2-specific), SR131675 (VEGFR3-specific), and motesanib diphosphate (pan-VEGFR inhibition), we determined that only VEGFR3 blockade significantly reversed MSC-mediated MRC proliferation (Extended Data Fig. 16A–C). Consistent with previous reports, VEGFA has the ability to modulate VEGFR3 heterodimerization or bind to the NRP2/VEGFR3 receptor complex, subsequently promoting VEGFR3 signaling activation [64–66]. Based on splenic stromal cell expression profiles (GSE274926), we revealed that MRCs express *Flt4* (the gene encoding VEGFR3), and immunofluorescence staining further confirmed VEGFR3 expression in MRCs (Extended Data Fig. 17A, B). Additionally, MSCs were in close proximity to VEGFR3^+^ MRCs, suggesting that MSCs likely exert their functional effects through VEGFA-mediated activation of VEGFR3 signaling in MRCs (Extended Data Fig. 17C, D). Therefore, MSCs may exert their functional effects through VEGFA-mediated activation of VEGFR3 receptor signaling on MRCs. To delineate the phosphorylation-dependent signaling cascades triggered by MSC-activated VEGFR3, we focused on key pathways linked to VEGFR activity, namely, the phosphoinositide 3-kinase (PI3K)–Akt pathway, and two mitogen-activated protein kinase (MAPK) pathway families have been characterized: classical MAPK (also known as extracellular signal-regulated kinase, Erk) and p38 kinase—both of which are critical for driving cellular proliferation and migration [67–69]. Splenic MRCs from aged mice were sorted and then cocultured in vitro with MSCs through a transwell system. We observed that the phosphorylation of Erk1/2 and Akt, rather than that of p38, was selectively induced in MRCs (Extended Data Fig. 18A). Consistent with these in vitro findings, intravenous administration of MSCs to aged mice also resulted in the phosphorylation of Akt and Erk1/2, but not p38, within splenic MRCs in vivo (Extended Data Fig. 18B–D). Moreover, this activation process was comparable to that of recombinant VEGFA stimulation (Extended Data Fig. 18E) and is critically dependent on VEGFR3 activity, as the VEGFR3 inhibitor SAR131615 markedly reduced MSC-induced phosphorylation (Extended Data Fig. 18F). Notably, the MSC-mediated proliferation of MRCs was significantly decreased by SCH772984 (a specific Erk1/2 inhibitor) or MK2206 (a selective Akt inhibitor), whereas Adezmapimod (a specific p38 inhibitor) had no significant effect (Extended Data Fig. 19A–E). Together, these results establish that ERK and Akt are the principal mediators of MSC-driven MRC proliferation. Collectively, these results indicate that VEGFA derived from MSCs binds to VEGFR3 on MRCs and primarily activates the downstream Erk and Akt signaling pathways, which in turn promote MRC expansion and stromal regeneration processes. These alterations ultimately facilitate the restoration of humoral immunity in aged models.

### MSC-mediated enhancement of vaccine efficacy was observed in aged cynomolgus monkeys

**Fig. 6:**
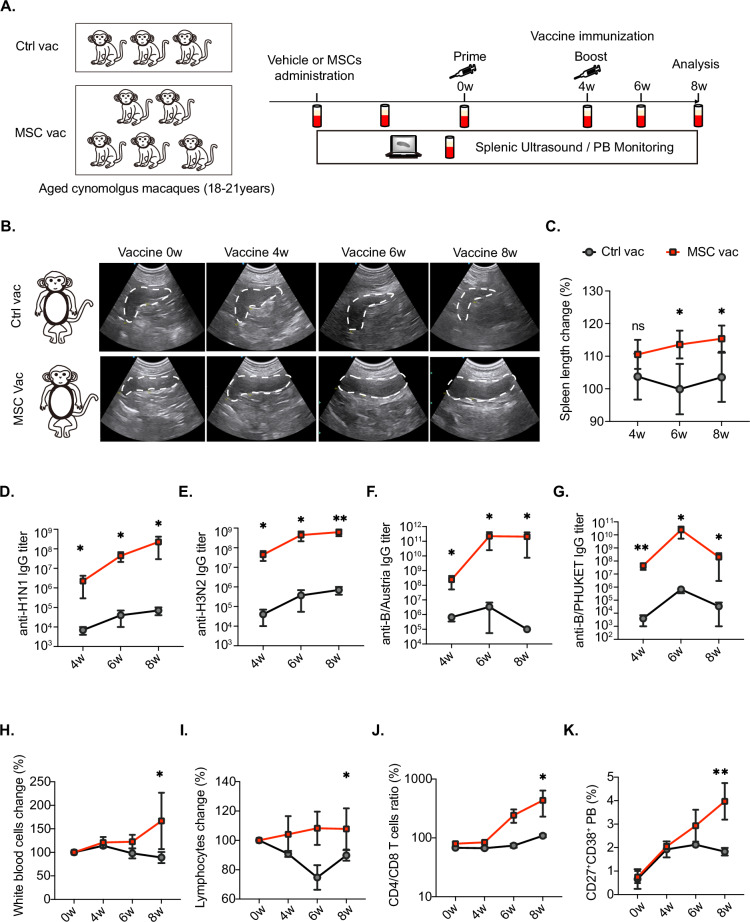
MSCs increase the level of the influenza vaccine in aged cynomolgus macaques. **A** Quadrivalent inactivated influenza vaccine immunization schedule in aged cynomolgus monkeys (18–21 years old) after vehicle (Ctrl vac, *n* = 3) or MSC vaccination for 9 weeks (MSC vac, *n* = 5). **B** Representative spleen ultrasound images after vaccination for 4, 6, and 8 weeks in the Ctrl vac and MSC vac groups. (**C**) Ultrasound measurement and analysis of spleen length changes in aged cynomolgus monkeys after vaccination for 4, 6, and 8 weeks in the Ctrl vac and MSC vac groups. Titers of serum vaccine-specific antigen (anti-recombinant H1N1 (**D**) anti-recombinant H3N2 (**E**) anti-recombinant B/PHUKET (**F**) and anti-recombinant B/Austria (**G**) soluble IgG antibodies after vaccination for 4, 6, and 8 weeks in the Ctrl vac and MSC vac. **H** Routine blood analysis of white blood cell changes in the aged Ctrl and aged+MSC groups. **I** Routine blood analysis of lymphocyte changes after vaccination for 4, 6, and 8 weeks in the Ctrl vac and MSC vac groups. **J** Peripheral blood lymphocyte population analysis of the ratio of CD4^+^/CD8^+^ T cells after vaccination for 4, 6, and 8 weeks in the Ctrl vac and MSC vac groups. **K** Peripheral plasma B-cell (CD19^+^CD27^+^CD38^+^) population analysis after vaccination for 4, 6, and 8 weeks in the Ctrl vac and MSC vac groups. The data are presented as the means ± SEMs of three or more independent experiments. Statistical significance was determined via a two-tailed unpaired *t-*test. *P < 0.05, **P < 0.01, ***P < 0.001, ****P < 0.0001. ns not significant

To assess the translational potential of MSC-based interventions for human vaccination strategies, we established an influenza vaccination model using aged cynomolgus monkeys (aged 17–20 years, Table 1), a species closely resembling humans in terms of immune system characteristics [70]. Baseline data, including blood samples and abdominal spleen ultrasonography data, were collected prior to MSC administration. Subsequently, MSCs or PBS (vehicle control) were intravenously delivered to aged cynomolgus monkeys, and daily health assessments were conducted (Fig. 6A). Nine weeks after MSC or vehicle administration, all the subjects received a primary immunization with 0.5 mL of quadrivalent influenza vaccine (QIV), which contained 15 µg of HA from each type of influenza strain (H1N1, H3N2, influenza B/Victoria lineage and influenza B/Yamagata lineage) [71]. Blood specimens and abdominal spleen ultrasonography were routinely conducted to monitor immune responses, and a booster inoculation was administered 4 weeks after the primary immunization (Fig. 6A). As delineated by abdominal ultrasound imaging, the immunological increase was correlated with an increase in splenic length, and the MSC-treated group (MSC+Vac, *n* = 5) presented a significant increase in splenic length at 2 and 4 weeks post-booster infection (Fig. 6B, C), whereas no notable changes were observed in the vehicle-infused group (Vac, *n* = 3). Moreover, vaccine-specific antibodies, including specific IgGs against influenza subtype A (H1N1 and H3N2) and B strains (Vitoria and PHUKET), were monitored before and after the booster dose. As anticipated, the MSC-administered group presented significantly higher titers of all four specific IgGs than did the vehicle-infused group after primary immunization (Fig. 6D–G). These titers were further elevated following the booster dose, particularly in the MSC-administered group (Fig. 6D–G). Importantly, the antibody levels in the MSC-administered group consistently surpassed those in the vehicle-infused group at all the corresponding time points (Fig. 6D–G), supporting the MSC-mediated enhancement of vaccine efficacy. In addition, blood analyses revealed an increase in the relative proportions of white blood cells and lymphocytes postimmunization, which were sustained for up to 8 weeks (Fig. 6H, I). The MSC-infused group also presented elevated lymphocyte counts, CD4/CD8 ratios, and CD19^+^CD27^+^CD38^+^ B cells (plasmablasts), further confirming a favorable vaccine response (Fig. 6J, K). These findings suggest that MSC therapy augments QIV vaccine responses in aged cynomolgus macaques, highlighting its potential to improve vaccine efficacy in elderly individuals.

**Table 1:** Cynomolgus monkey information

Number	Group	Sex	Age	Weight (kg)	Body condition scoring [93]
1	Sham	Male	20	6.06	3
2	Sham	Female	18	5.37	3
3	Sham	Female	19	3.82	2.5
4	MSC	Male	20	7.55	3
5	MSC	Female	20	4.81	3
6	MSC	Female	20	3.99	2
7	MSC	Female	19	3.39	1.5
8	MSC	Female	17	4.56	2.5

## Discussion

Vaccination remains the cornerstone of infection prevention in elderly individuals; however, age-related impairment of immune system functionality results in a decreased vaccine response [9]. In this study, we identified the aging splenic microenvironment as a critical limiting factor for GC formation and specific antibody generation. Notably, we demonstrated that MSC infusion prior to vaccine immunization effectively remodeled the splenic microenvironment governing the splenic MadCAM-1^+^ MRCs. Consequently, immune responses and antibody production are markedly enhanced postvaccination in aged mice and monkeys. Furthermore, the MSC-mediated increase in specific antibody production was primarily spleen dependent, as such effects were abolished in splenectomized mice (Extended Data Fig. 5). These findings provide proof of concept that the use of MSCs represents a novel strategy for modulating the aging splenic microenvironment to increase vaccine efficacy. Notably, MSCs administration in young mice failed to increase germinal center formation and OVA-specific antibody titers (data not shown), strongly suggesting that MSCs primarily restore age-impaired lymphoid niches rather than hyperstimulating competent immune systems.

The production of specific antibodies serves as a crucial mechanism for host defense against pathogens [72, 73], and various strategies, such as high-dose vaccines, multivalent formulations, and adjuvants, have been employed to improve vaccine responses [74]. While these approaches have shown partial success, the aging microenvironment poorly sustains or preserves plasma cells for long-term antibody production [25]. A growing body of evidence underscores the pivotal role of the aging microenvironment in limiting vaccine efficacy [23, 41, 75, 76]. Our study demonstrated that infused exogenous MSCs effectively localized to the spleen and remodeled its microenvironment in aged mice, leading to enhanced germinal center formation and increased specific antibody production. Additionally, our data suggest that MSCs are likely to regulate the splenic microenvironment through stromal cells rather than immune cells, further emphasizing the critical role of stromal cell modulation in reshaping the splenic niche.

As pivotal “custodians” of the cellular niche, splenic stromal cells play a critical role in establishing a supportive microenvironment, regulating immune cell trafficking, and facilitating their development [77]. However, during the aging process, the progressive decline in lymphoid tissue stromal cells contributes to the diminished immune response in older individuals [20, 78]. Consistent with these findings, we detected a significant reduction in the mature stromal cell population within the spleen. Importantly, we demonstrated that exogenous MSCs could restore the stromal cell network in aged mice by modulating the activation of splenic MAdCAM-1^+^ MRCs. This finding aligns with previous research indicating that PDGFRβ^+^MAdCAM-1^+^ stromal cells serve as essential progenitor cells in the development of the stromal cell network in the spleen [79]. MRCs are considered intermediate mesenchymal lymphoid tissue organizer (mLTo) cells capable of differentiating into follicular dendritic cells [80], which are crucial for supporting B-cell development and GC formation [81]. Our data corroborate this notion, as we detected MAdCAM-1^+^ cells in the white pulp region 3–4 weeks after MSC administration, accompanied by a gradual increase in FDC-M1^+^ FDC during the same period. These findings deepen our understanding of how exogenous MSCs modulate endogenous lymphoid tissue stromal cells to maintain immune homeostasis. Collectively, these insights highlight the potential of targeted MSC delivery to tissue-resident stromal cells or their precursor populations as a strategy to restore tissue homeostasis and achieve long-term therapeutic benefits.

Infused MSCs play critical roles in the maintenance, recovery, and regeneration of tissue homeostasis [31–33]. However, the underlying mechanism of MSC-mediated tissue homeostasis remains poorly understood. In this study, we identified VEGFA as a key functional molecule of MSCs that modulates MRC proliferation and restores the stromal cell network. A key component of this mechanism is VEGFR3, which we identified as a functionally relevant receptor on MRCs. Previous studies have implicated the VEGFA–VEGFR3/NRP2 axis in driving cellular proliferation [64–66], and we systematically demonstrated that phosphorylation of both the MAPK/Erk pathway and the PI3K/Akt pathway is essential for transducing VEGFA-mediated MRC activation. These findings provide novel insights into the role of VEGFA in the MSC-mediated regulation of splenic homeostasis and the rejuvenation of the aging spleen. VEGFA has broad implications in aging and tissue homeostasis, including its involvement in kidney injury repair, enhancement of pulmonary vascular repair following viral infections, and human skin rejuvenation [62, 82, 83]. Emerging evidence highlights the close association between VEGFA and aging processes, with VEGF signaling diminishing with age and VEGF supplementation demonstrating efficacy in promoting healthy aging [82]. Notably, both MSC-derived VEGFA and recombinant VEGFA effectively restored stromal network integrity in aged mice. We hypothesize that the reduced levels of VEGFA in aging individuals may impair the proliferation and differentiation of splenic stromal cells, akin to how low VEGFA levels disrupt myoblast differentiation and contribute to age-related muscle loss. These findings support the therapeutic potential of MSCs or VEGFA in addressing age-related immune decline, particularly in immune microenvironment remodeling, immune homeostasis regulation, and vaccine development. The mechanistic insights of this study not only elucidate a key signaling cascade in stromal crosstalk but also highlight the therapeutic potential for foundational repair of the immune architecture. While VEGFA administration partially mirrored the effects of MSCs, VEGFA represents one component of the pleiotropic activity of MSCs, and full therapeutic effects require multiple coordinated factors. Nonetheless, further research is needed to identify other candidates, develop combinatorial biomimetic formulations, and develop a spleen-targeted LNP delivery system to stimulate MRCs in the spleen.

In summary, our study offers compelling evidence that exogenous MSCs enhance the immune response and specific antibody production following vaccination in aged mice and monkeys and highlights the use of splenic MRCs as a promising strategy to restore the splenic architecture and bolster the immune response in aging individuals. These results significantly expand our understanding of the mechanisms of action of exogenous MSCs, in addition to their direct impacts on immune cells, to modulate the number and function of endogenous stromal cells and maintain immune homeostasis. Future investigations into the precise mechanisms by which exogenous MSCs govern tissue homeostasis will open new avenues for the clinical translation of cell-based therapies, offering potential strategies to combat age-related immune decline and enhance vaccine efficacy in older populations.

## Materials and methods

### Animal models

#### Mouse

Young female BALB/C mice (8–12 weeks) and aged female mice (>18 months) were purchased from Guangdong Yaokang Biotechnology Co., Ltd. (Guangdong, China). Female *Pdgfrb*-Cre mice (>10 months) were a gift from the laboratory of Professor Yamei Tang (Sun Yat-sen University). Female NDG (NOD. CB17-Prkdc scid Il2rg tm1 Bcgen/Bcgen) mice were purchased from BIOCYTOGEN Company (Beijing, China). *Madcam1*-CreERT2 mice were purchased from Shanghai Model Organisms (Shanghai, China). *ROSA26-iDTR* mice *were* purchased from The Jackson Laboratory (Maine, USA). All the mice were maintained under specific pathogen-free conditions at the Laboratory Animal Center of Sun Yat-sen University and maintained at a controlled temperature (24 °C ± 1 °C) and relative humidity (50% to 60%) with a 12-h (h) light/12-h dark cycle and provided standard rodent feed and water with ad libitum access. The mice were randomly assigned to experimental groups.

### Cynomolgus monkeys

Aged cynomolgus monkeys (aged 17–20 years) originating from Huazhen Biosciences (HZ-Bio) are housed in air-conditioned chambers with a maintained temperature (16–26 °C) and relative humidity (40–70%) with a 12 h light/12 h dark cycle and water, a commercially prepared primate diet, fresh fruits, and other supplements. Furthermore, toys, music, and other forms of entertainment and enrichment are supplied, and health is continuously monitored by a veterinarian with more than 20 years of experience. The detailed information of the cynomolgus monkeys is shown in Table 1.

### Ethics approval statement

All procedures followed the National Institutes of Health Guide for Care and relevant Sun Yat-Sen University guidelines and were approved by the Ethics Committee of Sun Yat-sen University (SYSU-IACUC-2024-B1562) and Guangzhou Huazhen Biosciences Company (HZ-EXF-001).

### MSC isolation

MSCs were derived from the bone marrow of healthy donors following the guidelines of the Helsinki Declaration, and informed consent was obtained, as described in our previous study [84–87]. Briefly, mononuclear cells were obtained via centrifugation via a density gradient with Ficoll-Hypaque (Amersham Biosciences, Cat#: 17544202) and then seeded at a density of 1 × 10^5^/cm^2^ in 75 cm^2^ culture flasks (Corning, CellBIND). When approximately 80% confluence was reached, the cells were detached with trypsin-EDTA and referred to as the first passage. Well-characterized 5th–8th passage MSCs were used for the experiments. A total of 1 × 10^6^ cells (passages 4–8) were suspended in 0.1 ml of PBS and transplanted into mice via tail vein injection for MSC administration. In cynomolgus monkeys, MSCs were suspended in 3–5 mL of normal saline at a dose of 2 × 10^6^ cells/kg body weight.

### Immunization

OVA (albumin from chicken egg white, grade II, Sigma, Cat# A5253) (100 ng) was dissolved in sterile ddH_2_O, and Freund’s adjuvant (Sigma, Cat# F5506) at a 1:1 ratio was used to immunize the mice intraperitoneally (i.p.). Booster immunizations were administered 14 days later via the intraperitoneal injection of 100 ng of OVA in mixed emulsification with incomplete Freund’s adjuvant (Sigma, Cat# F5881) [88]. Serological antibody levels were analyzed via ELISA, and germinal center reactions were analyzed on day 28 after the first immunization.

For influenza vaccine (HUALAN BIO, Quadrivalent/Inactivated/Split Virion) immunization, each mouse was injected intraperitoneally (i.p.) with 7.5 µg. Booster immunizations (7.5 µg) were administered 14 days after immunization. The serological vaccine antibody titer was detected via the hemagglutination inhibition test (HI), and the total serum sIgG antibody level was detected via ELISA. Germinal center reactions were analyzed by flow cytometry on day 28 after the first immunization.

For influenza vaccine (HUALAN BIO, Quadrivalent/Inactivated/Split Virion) immunization of cynomolgus monkeys, each monkey was injected intramuscularly (i.m.) with 15 µg. Booster immunizations (15 µg) were administered 28 days after immunization. Serological vaccine antibody titer and total serum sIgG antibody level detected by ELISA.

### Virus infection and sample collection

The animals were housed under SPF conditions with 12 h light and dark cycles before being transferred to the animal biosafety level 2 (ABSL2) laboratory for infection assays. Influenza virus A/Puerto Rico/8/34 (A/PR8/34, H1N1) was kindly provided by Jincun Zhao’s laboratory of the State Key Laboratory of Respiratory Disease and the National Clinical Research Center for Respiratory Disease in China. Fifty PFU of influenza virus A/PR8/34 (H1N1) were inoculated into the nasal cavity of the mice under isoflurane inhalation anesthesia. Each mouse received a total of 50 μL, with 25 μL inhaled into the left and right nostrils [89]. Weight loss was monitored daily after infection, and the mice were humanely euthanized when 25% of their body weight was lost. The mice were sacrificed on day 14 postinfection, and lung, spleen, and blood samples were collected. Blood samples were centrifuged at 2600 rpm for 25 min at 4 °C in the presence of heparin sodium, and the supernatant was collected as the plasma. Lungs and spleens were fixed in 4% paraformaldehyde until further processing. The clinical score criteria included the presence and extent of inflammatory infiltrates, edema, hyperemia, and congestion, and focal necrosis of the alveolar epithelium. The scoring criteria were as follows: 0 for no changes, 1 for mild changes, 2 for moderate changes, 3 for marked changes, and 4 for severe changes [90]. Animal husbandry and use and experimental procedures were in accordance with national guidelines and were approved by the Animal Husbandry and Use Committee.

### Diphtheria toxin (DT)-mediated splenic stromal cell ablation

To ablate splenic PDGFRβ^+^ cells and ensure the specificity of the system, *Pdgfrb*-Cre mice were injected directly with AAV-DTR (PAV-CAG-DIO-DTR-P2A-mCherry) at a dose of 1 × 10^11^ viral genomes (vg) per mouse. After 10 days, the AAV-injected mice received three intraperitoneal injections of 100 ng diphtheria toxin (Millipore, Cat#: 322326) or saline, which were administered only once daily [91].

### Splenic cell isolation

Stromal cells were released by enzymatic digestion essentially as previously described [92]. Freshly obtained mouse spleens were treated with collagenase type IV (2 mg/ml, Gibco, Cat#: 17104019), DNase I (0.1 mg/ml, Thermo Scientific, Cat#: EN0521), Liberase (0.2 mg/ml, Roche, Cat#: 5401119001) and Dipasase (0.8 mg/ml, Roche, Cat#: 4942078001). The splenocyte suspension was obtained via effective and gentle treatment of the spleen tissue via a gentle MACS Dissociator (Miltenyi Biotec, Cat#: 130-093-235). Then, they were put into a 37 °C water bath for digestion, which was frequently agitated during digestion. The cells were harvested and suspended in a-MEM containing 1% FBS (PAN-Biotech, Cat#: P30-3033) and 2 mM EDTA (Sigma‒Aldrich, Cat#: 4008-M). The cells were filtered through a 70 µm pore size filter. Individual cells were collected and washed three times with PBS. Red blood cells were removed via red cell lysis buffer (Roche). Splenocytes were resuspended in a-MEM (HyClone, Cat#: SH30265. FS) supplemented with 10% FBS and 1% penicillin/streptomycin (HyClone, Cat#: SV30010) to maintain cell viability. Spleen stromal cells (CD45^-^CD31^-^Ter119^-^PDGFRβ^+^) were sorted via flow cytometry.

### Flow cytometry

The cells were collected into flow tubes and centrifuged at 1500 rpm for 5 min to obtain cell precipitates. The cells were washed twice with phosphate-buffered saline (PBS; Gibco, Cat#: 10010023). Each sample was diluted and resuspended in perm buffer: antibody = 50:1 (VOL) and incubated for 30 min at 4 °C in the dark. The cells were washed twice with PBS and centrifuged at 1500 rpm for 5 min. The cells were then resuspended in PBS, and the cell suspension was filtered through a 70 μm cell strainer (Corning, Cat#: 352350) and analyzed by flow cytometry. The antibodies used were as recommended by the manufacturer and are described in the Supplementary Information (Table S1). The data were examined with a CytoFLEX flow cytometer (Beckman) and analyzed with FlowJo 10.6.2 software (FlowJo LLC).

### Immunofluorescence

Freshly harvested mouse spleens were fixed with 4% paraformaldehyde (PFA, PHYGENE, Cat#: PH0427) overnight at 4 °C and then dehydrated in 30% sucrose (Millipore Sigma, Cat#: 57--50-1) solution at 4 °C until the tissues sank. The prepared spleens were then embedded in Tissue-Tek optimum cutting temperature compound (Sakura, Cat#: 4583) and stored at −80 °C until 15 μm sections were processed on a cryotome (RWD FS800). Spleen tissue sections were incubated with goat serum (BOSTER, Cat#: AR0009) for 30 min after incubation with 0.5% Triton X-100 (Sigma Aldrich, Cat#: 9036-19-5) for 20 min at room temperature. For staining, primary antibodies (diluted in PBS containing 0.1% BSA) were used to incubate the sections overnight at 4 °C. After 5 washes, the sections were then incubated with secondary antibodies (diluted in PBS containing 0.1% BSA) for 2 h at room temperature. Antibodies were used as recommended by the manufacturer, and the details are provided in the Supplementary Information (Table S1). Additionally, DAPI (Sigma Aldrich, Cat#: D9542) was used to identify the cell nucleus. The stained sections were mounted in fluorescence mounting medium (Dako, Cat#: 302380-2) with glass coverslips (CITOTEST, Cat#: 0212450C) and imaged with confocal microscopy (Dragonfly, CR-DFLY-202 2540).

### Histological examination

Formalin fixation, paraffin embedding, and sectioning were used to prepare mouse spleen, lymph node, and lung tissues. In brief, 4 μm thick sections were cut from formalin-fixed, paraffin-embedded tissue for each assay. The slides were deparaffinized, rehydrated, and boiled at 97 °C in citrate buffer (pH 6) for 20 min to extract epitopes. Endogenous peroxidase was then blocked by incubation with 3% H_2_O_2_ for 15 min, and the tissue sections were covered with blocking buffer for 10 min at room temperature. The samples were subsequently stained with hematoxylin and eosin (Sigma Aldrich, Cat#: H9627 and Cat#: E4009).

### ELISA

Plasma or alveolar lavage fluid from normal control and model mice was collected into tubes and centrifuged at 2500 rpm/min for 20 min at 4 °C, after which the supernatant was aspirated for analysis. ELISA kits for mouse IgG, IFN-γ, and TNF-α were used (Neobioscience, Cat# EMC116, EMC101g, and EMC102a). OVA-sIgG1 was detected in the mice via a Mouse Anti-OVA IgG1 Antibody Assay Kit (Chondrex, Cat#: 3013). The experiments were performed according to the reagent manufacturer’s instructions. Briefly, the collected supernatants were added to ready-made 96-well plates coated with antibodies against TNF-α, IL-1β, IFN-γ, soluble OVA-IgG1, and IgG, which were assayed via the sandwich ELISA technique. Finally, the absorbance of each well was determined with a microplate reader (Thermo Scientific).

For influenza vaccine (HUALAN BIO, Quadrivalent/Inactivated/Split Virion)-specific antibodies, every recombinant antigen HA protein, including influenza A H1N1 (A/Victoria/4897/2022) hemagglutinin, influenza A H3N2 (A/Darwin/9/2021) hemagglutinin, influenza B (B/Austria/1359417/2021) hemagglutinin and influenza B (B/PHUKET/3073/2013) hemagglutinin, which were all purchased from Sino Biological Company, was diluted in coating buffer (1 μg/ml), added to a 96-well clear flat bottom polystyrene high-binding microplate (Corning, Cat#: 9018) and incubated for 16 h at 4 °C. The wells were washed with 300 μL of wash buffer five times and blocked with 200 μL of PBST  +  5.0% nonfat dry milk (blocking buffer) for 2 h at room temperature. The plate was then washed three times with 300 μL of wash buffer. The plates were incubated with horseradish peroxidase (HRP)-conjugated goat anti-monkey IgG (1:10,000, Invitrogen) for 60 min at 37 °C. The plates were then washed five times with wash buffer, and chromogen solution was added, followed by 15 min of incubation at 37 °C. The absorbance (450/630 nm) was read via a microplate reader (BioTek). The endpoint titers were defined according to the manufacturer’s instructions.

### Western blot

To evaluate the impact of VEGFA siRNA on VEGFA protein levels in MSCs, Western blotting experiments were conducted. At 72 h postsiRNA transfection, we collected culture medium from the MSCs and lysed the adherent cells with RIPA lysis buffer (Sigma‒Aldrich, Cat#: R0278) containing PMSF (Roche, CAS: 329-98-6) for protein extraction. The protein concentration was determined via the Pierce™ BCA Protein Assay Kit (Thermo Fisher Scientific, Cat#: 23227). After denaturation at 100 °C, a standard WB procedure provided by Abcam was used to assess the protein expression levels of VEGFA and GAPDH. SDS‒PAGE electrophoresis, membrane transfer, blocking, and incubation with primary (anti-human VEGFA, Abcam, Cat# ab46154; GAPDH, Cell Signaling, Cat# 97166) and secondary (anti-rabbit IgG HRP-linked, Cell Signaling, Cat# 7074; anti-mouse IgG HRP-linked, Cell Signaling, Cat# 7076) antibodies were performed with 10 µg of total protein in the supernatant and total protein in the cell lysate. Finally, we detected protein signals via chemiluminescence and imaged them via a ChemiDoc imaging system (Bio-Rad).

To evaluate the phosphorylation of Erk1/2, Akt, and p38 in MRCs, Western blotting experiments were conducted. At 72 h post-culture, the cells were cultured with or without MSCs in a transwell system in vitro. The small molecule inhibitors used were as follows: SAR131675 (VEGFR3 inhibitor, Selleck, Cat#: S2842), SCH772984 (Erk1/2 inhibitor, Selleck, Cat#: S7101), MK2206 (Akt inhibitor, Selleck, Cat#: S1078), and Adezmapimod (p38 inhibitor, Selleck, Cat#: S1076). We collected MRCs and lysed adherent cells via RIPA lysis buffer (Sigma‒Aldrich, Cat#: R0278) containing phenylmethylsulfonyl fluoride (PMSF, Roche, CAS: 329-98-6) and a phosphatase inhibitor cocktail (MedChemExpress, Cat#: HY-K0022) for protein extraction. The protein concentration was determined via the Pierce™ BCA Protein Assay Kit (Thermo Fisher Scientific, Cat#: 23227). After denaturation at 100 °C, a standard WB procedure provided by Abcam was used to assess the protein expression levels of phosphorylated (p)-AKT, p-Erk, p-p38, pan-Akt, Erk, p38, and β-actin. SDS‒PAGE electrophoresis, membrane transfer, blocking, and incubation with primary antibodies (phospho-Erk1/2 (Thr202/Tyr204), Cell Signaling, Cat#4370; phospho-Akt (Ser473), Cell Signaling, Cat#4060; phospho-p38 (Thr180/Tyr182), Cell Signaling, Cat#4511; Erk1/2, Cell Signaling, Cat#8690; Akt (pan), Cell Signaling, Cat#4691; Erk1/2, Cell Signaling, Cat#4696; p38, Cell Signaling, Cat#8690; β-Actin, Cell Signaling, Cat# 4970; α-Tubulin, Cell Signaling, Cat#3873) and secondary antibodies (anti-rabbit IgG HRP-linked, Cell Signaling, Cat#7074; anti-mouse IgG HRP-linked, Cell Signaling, Cat# 7076) were performed with 10 µg of total protein in the supernatant and total protein in the cell lysate. Finally, we detected protein signals via chemiluminescence and imaged them via a ChemiDoc imaging system (Bio-Rad).

### RNA-seq and analysis

Utilizing the Ensembl database GRCh38, filtering was conducted via fastp (v0.23.4), followed by alignment with STAR (2.7.11b) and quantification via RSEM (V1.3.3) to obtain raw counts and TPM for the MSC samples. Bulk RNA-seq data of mouse spleen MRCs were sourced from GSE171124, and TPM was calculated on the basis of gene length referencing GRCm39. Predictive Analysis of Cell Communication: Receptor‒ligand Interactions between Mouse Spleen MRCs and Human Bone Marrow MSCs. Initially, mouse genes were converted to their human homologs via the R package biomaRt (v2.58.0), followed by filtering of genes expressed at low levels via the R package edgeR (v4.0.16). Receptor genes were extracted from the CellChat database (v2.1.2), and an intersection was taken between the spleen MRC genes and the database’s receptor genes, resulting in 319 receptor genes. To narrow down the scope, KEGG enrichment analysis of receptor genes was conducted via the R package clusterProfiler (v4.10.0), which retained only pathways relevant to proliferation. Subsequently, 67 receptor genes were obtained and sorted on the basis of their expression levels, and a heatmap was generated via the R package pheatmap (v1.0.12). The corresponding ligand genes were identified by comparison with the database, followed by subset selection from the MSC expression matrix, resulting in 40 ligand genes, which were visualized in the heatmap.

### Statistics and reproducibility

The experiments described in the study were repeated a minimum of three times to ensure reliability and reproducibility. The figure legends indicate the combination of data from independent experiments. Data analysis and visualization were conducted via Prism 9 (GraphPad) software. Statistical analysis was performed via one-way analysis of variance (ANOVA), the Kruskal‒Wallis multiple comparison test, the Mann‒Whitney rank sum test, or the multiple unpaired *t*-test, as specified in the figure legends. A significance level of *P* < 0.05 was considered statistically significant. Each data point in the figures represents a biological replicate, whereas the line represents the median value.

## Supplementary information

Supplementary Information (Table S1)

Extended Data Figure Legends

## Source data

Source Figure data
