# Supplementary material for: Mesenchymal stromal cells counteract with age-related immune decline and enhance vaccine efficacy by modulating endogenous splenic marginal reticular cells in elderly models
Source: Cell Mol Immunol. 2026 Jan 9;23(2):220–35. doi: 10.1038/s41423-025-01381-9 (PMC12858895; doi:10.1038/s41423-025-01381-9)
Supplement: Supplementary file 1 — Supplementary Information (Table S1) [file 41423_2025_1381_MOESM1_ESM.docx]

**Table S1. Antibody**

| Antibody | Clone | Manufactory |  |
| --- | --- | --- | --- |
| Anti-Μouse CD45 | 30-F11 | BD Biosciences |  |
| Anti-Μouse CD31 | 390 | Biolegend |  |
| Anti-Mouse TER-119 | TER-119 (RUO) | BD Biosciences |  |
| Anti-Mouse CD140B (PDGFRβ) | APB5 [(RUO)](https://www.bdbiosciences.com/zh-cn/products/reagents/flow-cytometry-reagents/research-reagents/single-color-antibodies-ruo/pe-rat-anti-mouse-cd140b-pdgfr.569797) | BD Biosciences | |
| Anti-Mouse B220 | RA3-6B2 | Biolegend/BD Biosciences | |
| Anti-Mouse CD3e① | 17A2 | Biolegend | |
| Anti-Mouse CD3e② | 145-2C11 | BD Biosciences | |
| Anti-Mouse CD4 | GK1.5 | Biolegend | |
| Anti-Mouse CD8 | 53-5.8 | Biolegend | |
| Anti-Mouse GL7 | GL7 | Biolegend | |
| Anti-Mouse IgD | 11-26c.2a | Biolegend | |
| Anti-Mouse CD157 | BP-3 (RUO) | BD Biosciences | |
| Anti-Mosue Ki-67 | SP6 | Thermo Fisher | |
| Anti-Mosue Ki-67 | 16A8 | Biolegend | |
| Anti-Mouse MAdCAM-1① | H-3 | Santa Cruz Biotechnology | |
| Anti-Mouse MAdCAM-1② | MECA-367 | Biolegend | |
| Anti-Mouse PDPN | eBio8.1.1 | eBioscience | |
| Anti-Mouse CD21/35 | 7E9 | Biolegend | |
| Anti-Human VEGFA | P15692 | Abcam | |
| Anti- Phospho-Erk1/2 | D13.14.4E | CellSignaling | |
| Anti- Phospho-Akt | D9E | CellSignaling | |
| Anti- Phospho-p38 | D3F9 | CellSignaling | |
| Erk1/2 | L34F12 | CellSignaling | |
| Akt | C67E7 | CellSignaling | |
| p38 | D13E1 | CellSignaling | |
| GAPDH | D4C6R | CellSignaling | |
| β-Actin | 13E5 | CellSignaling | |
| α-Tubulin | DM1A | CellSignaling | |
| Anti-rabbit IgG HRP-linked | Heavy and light chain | CellSignaling | |
| Anti-mouse IgG HRP-linked | Heavy and light chain | CellSignaling | |
| Anti-Monkey IgG (H+L) Secondary Antibody, HRP | IgG (H+L) | Invitrogen | |
| Anti-NHP CD45 | D058-1283 | BD Biosciences | |
| Anti-Human CD3 | SP34-2 | BD Biosciences | |
| Anti-Human CD4 | L200 | BD Biosciences | |
| Anti-Human CD19 | HIB19 | BD Biosciences | |
| Anti-Human CD27 | M-T271 | BD Biosciences | |
| Anti-Human CD38 | HIT2 | BD Biosciences | |
