# Supplementary material for: Mesenchymal stromal cells counteract with age-related immune decline and enhance vaccine efficacy by modulating endogenous splenic marginal reticular cells in elderly models
Source: Cell Mol Immunol. 2026 Jan 9;23(2):220–35. doi: 10.1038/s41423-025-01381-9 (PMC12858895; doi:10.1038/s41423-025-01381-9)
Supplement: Supplementary file 2 — Extended Data Figure Legends [file 41423_2025_1381_MOESM2_ESM.docx]

**Extended Data Figure Legends**

**Extended Data Fig. 1. Diminished Splenic Stromal Cells and Architectural Defects in Aged Mice.**

(A) Representative gross observations of aged (>18-month-old) BALB/c spleens from young (2–3 months) and aged (>18-month-old) mice. (B) Statistical analysis of the spleen weight/body weight ratio in the young and aged groups; n=5 mice per group. (C) Statistical analysis of spleen cell counts in the young and aged groups; n=5 mice per group. (D) Representative flow cytometry results of the proportion of splenic stromal cells (CD45^-^CD31^-^Ter119^-^PDGFRβ^+^) in the young and aged groups. (E) Statistical analysis of the proportion of splenic stromal cells (CD45^-^CD31^-^Ter119^-^PDGFRβ^+^) in the young and aged groups; n=5 mice per group. (F) Statistical analysis of splenic stromal cell counts in the young and aged groups; n=5 mice per group. (G) Representative immunofluorescence staining of splenic stromal cells (PDGFRβ^+^, fuchsia) in the young and aged groups. (H) Statistical analysis of the splenic stromal cell area in the young and aged groups; n=5 mice per group. (I) Representative immunofluorescence staining of splenic T lymphocytes (CD3e^+^, green) and B lymphocytes (B220^+^, red) in the young and aged groups. (J) Representative H&E staining of spleens from the young and aged groups. The data represent the means ± SEMs of 3 or more independent experiments. Statistical significance was determined via a two-tailed unpaired t test. *P < 0.05, **P < 0.01, ***P < 0.001, ****P < 0.0001. ns, not significant.

**Extended Data Fig. 2. MSCs promote splenic stromal cell architecture and lymphocyte expansion in aged mice.**

(A) Representative immunofluorescence staining of splenic stromal cells (PDGFRβ^+^, fuchsia) in the control (Vehicle), HDF-treated 28-day (HDF), and MSC-treated 28-day (MSC) groups; scale bars: 150 μm. (B) Statistical analysis of the splenic stromal cell area in the vehicle, HFD, and MSC groups; n = 5 mice per group. (C) Statistical analysis of the mean fluorescence intensity (MFI) of splenic stromal cells in the vehicle, HFD, and MSC groups; n = 5 mice per group. (D) Representative immunofluorescence staining of splenic T lymphocytes (CD3e^+^, green) and B lymphocytes (B220^+^, red) in the vehicle, HDF, and MSC groups; scale bars: 100 μm. (E-F) Statistical analysis of the B lymphocyte area and T lymphocyte area in the vehicle and 1--4-week-old MSC groups; n = 5 mice per group. The data represent the means ± SEMs of 3 or more independent experiments. Statistical significance was determined via one-way ANOVA with multiple comparisons. *P < 0.05, **P < 0.01, ***P < 0.001, ****P < 0.0001. ns, not significant.

**Extended Data Fig. 3. Residence of MSCs in the spleen.**

(A) Representative immunofluorescence images of MSCs (RFP^+^) and DAPI (blue) in the groups at 1, 2, 3, and 4 weeks after MSC infusion. (B) MSCs distributed in the spleen, liver and kidney in vivo.

**Extended Data Fig. 4. MSCs promoted aged spleen germinal center formation after OVA immunization.**

(A) Colocalization between follicular B (FOB) cell clusters, T follicular helper (TFH) cell clusters and GCs. Follicular B cells cluster (IgD^+^, red), T follicular helper cells (CD4^+^ in GC, green), and germinal center B cells (GL-7^+^, white); scale bar: 50 μm.

**Extended Data Fig. 5. The spleen is an important target tissue for MSCs to increase the age-related immune response.**

(A) Schematic diagram of the splenectomy mouse model, MSC infusion and immunization. (B) Concentrations of serum soluble ovalbumin-specific IgG1 antibodies 28 days after immunization in the aged sham, aged + MSC, aged splenectomy and aged splenectomy + MSC groups. (C) Representative flow cytometry of plasma cells (gated on B220^+^ and CD138^+^) in the aged sham, aged + MSC, aged splenectomy and aged splenectomy + MSC groups. (D) Proportion of plasma cells in the aged sham, aged + MSC, aged splenectomy and aged splenectomy + MSC groups. The data represent the means ± SEMs of 3 or more independent experiments. Statistical significance was determined via one-way ANOVA with multiple comparisons. *P < 0.05, **P < 0.01, ***P < 0.001, ****P < 0.0001. ns, not significant.

**Extended Data Fig. 6. MSCs restore spleen microarchitectures mainly by modulating stromal cells.**

(A) Representative images of immunofluorescence staining of splenic stromal cells (PDGFRβ^+^, fuchsia) in the NDG control and MSC-treated groups at 28 days (NDG+MSC); scale bars: 124.5 μm. (B) Representative immunofluorescence staining of DAPI (blue), splenic T lymphocytes (CD3e^+^, red) and B lymphocytes (B220^+^, green) in the groups of splenic stromal cell depletion control (DTR) and MSC-treated (DTR+MSCs); scale bars: 500 μm.

**Extended Data Fig. 7. The PDGFRβ-cre × AAV-iDTR system was established to selectively deplete splenic stromal populations.**

(A) An adeno-associated virus (AAV) carrying a Cre-dependent construct (PAV-CAG-DIO-DTR-P2A-mCherry) system to selectively deplete splenic stromal cells. (B) The splenic stromal cell population in the Ctrl AAV and AAV-DTR groups was detected by flow cytometry. (C) Representative immunofluorescence images were used to detect PDGFRβ^+^ cells in the spleens of the Ctrl AAV and AAV-DTR groups; scale bar: 500 µm. (D) Representative immunofluorescence images were used to detect PDGFRβ^+^ cells in the livers of the Ctrl AAV and AAV-DTR groups; scale bar: 10 µm.

**Extended Data Fig. 8. MSCs cannot produce specific antibodies in mice with specific spleen stromal cell deficiency.**

(A) AAV-DTR injection specifically cleared splenic stromal cells, followed by MSC infusion and OVA immunization in the control AAV (OVA control), control AAV and MSC (MSC+OVA), AAV-DTR control (DTR OVA) and AAV-DTR and MSC (DTR+MSC OVA) groups. (B) Titer of total serum IgG1 antibodies 28 days after immunization in the OVA control, MSC+OVA, DTR OVA and DTR+MSC OVA groups. (C) Representative flow cytometry of splenic germinal center B-cell populations (B220^+^IgD^-^GL7^+^) in the OVA control, MSC+OVA, DTR OVA and DTR+MSC OVA groups. (D) Statistical analysis of the proportions of splenic germinal center B cells in the OVA control, MSC+OVA, DTR OVA and DTR+MSC OVA groups; n=3 mice per group. (E) Representative images of immunofluorescence staining of follicular B lymphocytes (IgD^+^, red) and germinal centers (PNA^+^, green) in the OVA control, MSC+OVA, DTR OVA and DTR+MSC OVA groups. (F) Representative flow cytometry of splenic stromal cells (CD45^-^CD31^-^Ter119^-^PDGFRβ^+^) in the OVA control, MSC+OVA, DTR OVA and DTR+MSC OVA groups. (G) Statistical analysis of the proportion of splenic stromal cells in the OVA control, MSC+OVA, DTR OVA and DTR+MSC OVA groups; n=3 mice per group. (H) Representative images of immunofluorescence staining of splenic stromal cells (PDGFRβ^+^ and fuchsia) in the OVA control, MSC+OVA, DTR OVA and DTR+MSC OVA groups. The data represent the means ± SEMs of 3 or more independent experiments. Statistical significance was determined via one-way ANOVA with multiple comparisons. *P < 0.05, **P < 0.01, ***P < 0.001, ****P < 0.0001. ns, not significant.

**Extended Data Fig. 9. MSCs promote splenic cell proliferation in aged mice, particularly CD45- cells.**

(A) Flow cytometry analysis of the proportion of proliferating splenic cells (KI-67^+^) in the vehicle (Aged Ctrl) or MSC (Aged + MSC) groups after 3 days; n=3 mice per group. (B) Flow cytometry analysis of the proportion of proliferating splenic immune cells (CD45+KI-67^+^) in the vehicle (Aged Ctrl) or MSC (Aged + MSC) groups after 3 days; n=3 mice per group. (C) Flow cytometry analysis of the proportion of proliferating splenic structural cells (CD45^-^KI-67^+^) in the vehicle (Aged Ctrl) or MSC (Aged + MSC) groups after 3 days; n=3 mice per group. (D) Statistical analysis of the ratio of splenic cell proliferation in the MSC-treated group to the aged Ctrl group in the groups of CD45^-^ and CD45^+^ splenic cells; n=3 mice per group. The data represent the means ± SEMs of 3 or more independent experiments. Statistical significance was determined via a two-tailed unpaired t test. *P < 0.05, **P < 0.01, ***P < 0.001, ****P < 0.0001. ns, not significant.

**Extended Data Fig. 1****0. Spatial proximity between MSCs and MRCs promotes KI-67 expression in MRCs**

(A) Representative immunofluorescence staining of KI-67 (green) expression in splenic MRCs (MAdCAM-1^+^, fuchsia) in the vehicle- and MSC-treated groups. (B) Representative fluorescence staining of MRCs (KI-67⁺; KI-67⁻) and their distance from MSCs. (C) Statistical analysis of MRCs (KI-67⁺; KI-67⁻) and their distance from MSCs. The data represent the means ± SEMs of 3 or more independent experiments. Statistical significance was determined via a two-tailed unpaired t test. *P < 0.05, **P < 0.01, ***P < 0.001, ****P < 0.0001. ns, not significant.

**Extended Data Fig. 11. Limited impact of MSCs on the proliferation of splenic FDC and FRC cells in aged mice**

(A) Representative immunofluorescence staining of splenic follicular dendritic cell proliferation (FDC-M1^+^, blue, KI-67^+^, green and DAPI, gray) and analysis of the vehicle (Aged Ctrl) or MSC (Aged + MSC) groups after 3 days; n=5 mice per group. (B) Representative immunofluorescence staining of splenic follicular reticular cell proliferation (PDPN^+^, orange, KI-67, green and DAPI, gray) and analysis of the groups treated with vehicle (Aged Ctrl) or MSC (Aged + MSC) for 3 days; n=5 mice per group. The data represent the means ± SEMs of 3 or more independent experiments. Statistical significance was determined via a two-tailed unpaired t test. *P < 0.05, **P < 0.01, ***P < 0.001, ****P < 0.0001. ns, not significant.

**Extended Data Fig. 12. Limited impact of MSCs on the proliferation of splenic FDCs and FRCs in aged mice.**

(A) Generation of genetic mice with tamoxifen-induced diphtheria toxin conditional ablation of MRCs (*Madcam-1 cre/ERT2* × *ROSA26 iDTR*). (B) Tamoxifen induced diphtheria toxin conditional ablation in *Madcam-1 cre/ERT2* x *ROSA26 iDTR* mice and *Madcam-1 cre/ERT2* mice as nonablation controls. (C) The ablation effect on MRCs in the spleen was detected by flow cytometry (CD45^-^CD31^-^Ter119^-^MAdCAM-1^+^). (D) Representative immunofluorescence results were used to detect MRCs (MAdCAM-1^+^, red) and FDCs (FDC-M1^+^, green) from spleens in the *Madcam-1 cre/ERT2* and *Madcam-1 cre/ERT2* × *ROSA26 iDTR groups.*

**Extended Data Fig. 13. Following MRC deficiency, the ability of MSCs to promote splenic germinal center formation and enhance OVA-specific antibody production was abolished.**

(A) OVA immunization procedure after MRC ablation. (B) Ablation of MRCs in the spleen was detected by flow cytometry (CD45^-^CD31^-^Ter119^-^MAdCAM-1^+^). (C) Representative immunofluorescence results were used to detect MRCs (MAdCAM-1^+^, red) in the spleens (scale bar: 50 µm)*.* (D) Representative immunofluorescence results were used to detect GCs (GL-7^+^, white), FOB (IgD^+^, red) and Tfhs (CD4^+^ colocalized in GCs, green); scale bar: 50 µm. (E) Concentrations and titers of serum soluble ovalbumin-specific IgG1 antibodies 28 days after immunization.

**Extended Data Fig. 14. VEGFA is a Potential Functional Molecule of MSCs**

(A) The top 67 receptor molecules associated with cell proliferation in spleen MRCs were ranked on the basis of their expression levels. (B) Ligand molecules corresponding to (A) were screened and ranked via bulk RNA sequencing data from naïve MSCs in vitro and in aged spleens.

**Extended Data Fig. 15. Impaired Function of MSCs in Promoting Splenic MRC Proliferation and Spleen Stromal and lymphocyte Architecture Recovery in Aged Mice Due to Defective Expression of VEGFA**

(A) VEGFA protein expression in the MSC*^NC,^* MSC*^VEGFA-KD1^*, MSC*^VEGFA-KD2^*, and MSC*^VEGFA-KD3^* groups was detected via Western blotting. (B) Relative VEGFB, VEGFC and VEGFD mRNA expression detected through RT‒PCR validation in the MSC*^NC^* and MSC*^VEGFA-KD groups^*, n=3. (C) Vehicles (vehicle), nonsense control MSCs (MSC*^NC^*) or VEGFA-knockdown MSCs (MSC*^VEGFA-KD^*) were administered to aged BALB/c mice (>18 months old), which were analyzed 3 days later. (D) Representative immunofluorescence staining of splenic MRCs (MAdCAM-1^+^, fuchsia), KI-67 (green) and DAPI (gray) in the vehicle, MSC*^NC^* and MSC*^VEGFA-KD^* groups; scale bars: 40 μm. (E) Vehicles (vehicle), nonsense control MSC (MSC*^NC^*) or VEGFA-knockdown MSC (MSC*^VEGFA-KD^*) were administered to aged BALB/c mice (>18 months old), which were analyzed 30 days later. (F) Statistical analysis of the spleen weight/body weight ratio and splenic cell count in the vehicle, MSC*^NC^* and MSC*^VEGFA-KD groups^*. (G) Representative immunofluorescence staining of splenic T lymphocytes (CD3e^+^, green) and B lymphocytes (B220^+^, red) in the vehicle, MSC*^NC^* and MSC*^VEGFA-KD^* groups; scale bars: 300 μm. The data represent the means ± SEMs of 3 or more independent experiments. Statistical significance was determined via one-way ANOVA with multiple comparisons. *P < 0.05, **P < 0.01, ***P < 0.001, ****P < 0.0001. ns, not significant.

**Extended Data Fig. 16. VEGFR3 blockade significantly reversed MSC-mediated MRC proliferation.**

(A) Schematic diagram of sorting of splenic CD45^-^CD31^-^Ter119^-^MadCAM-1^+^ cells. (B) Representative plots of Ki-67 expression in MRCs from the MRC control, MRC + MSC, MRC+MSC+VEGFR1 inhibitor, MRC+MSC+VEGFR2 inhibitor, MRC+MSC+VEGFR3 inhibitor and MRC+MSC+VEGFR inhibitor groups. (C) Analysis of Ki-67 expression in MRCs from the MRC control, MRC + MSC, MRC+MSC+VEGFR1 inhibitor, MRC+MSC+VEGFR2 inhibitor, MRC+MSC+VEGFR3 inhibitor and MRC+MSC+VEGFR inhibitor groups. The data represent the means ± SEMs of 3 or more independent experiments. Statistical significance was determined via one-way ANOVA with multiple comparisons. *P < 0.05, **P < 0.01, ***P < 0.001, ****P < 0.0001. ns, not significant.

**Extended Data Fig. 17. Splenic MRCs express VEGFR3**

(A) Violin plots of FIt4 mRNA expression in spleen stromal subsets; data from GSE274926. (B-C) Representative fluorescence images of VEGFR3 (green) expression by MRCs (MAdCAM-1^+^, fuchsia) in the control and MSC (red) groups. (D) Representative 3D modeling diagram illustrating the spatial distribution of VEGFR3⁺ (green) MRCs (MAdCAM-1^+^, fuchsia) in close proximity to MSCs (red) via fluorescence staining.

**Extended Data Fig. 18. MSCs promote the phosphorylation of Akt/Erk in splenic MRCs through the VEGFA-VEGFR3 axis**

(A) Representative western blot analysis of Erk1/2 phosphorylation (Thr202/Tyr204), Akt phosphorylation (Ser473) and p38 phosphorylation (Ser473) in splenic MRCs from two groups: MRC control and MSC-transwell cocultures. (B) Representative immunofluorescence image showing Erk1/2 phosphorylation (Thr202/Tyr204, turquoise) in splenic MRCs (MAdCAM-1^+^, fuchsia) from mice treated with vehicle or MSCs. (C) Representative immunofluorescence image showing Akt phosphorylation (Ser473, turquoise) in splenic MRCs (MAdCAM-1^+^, fuchsia) from mice treated with vehicle or MSCs. (D) Representative immunofluorescence image showing p38 phosphorylation (Ser473, turquoise) in splenic MRCs (MAdCAM-1^+^, fuchsia) from mice treated with vehicle or MSCs. (E) Representative western blot analysis of Erk1/2 phosphorylation (Thr202/Tyr204), Akt phosphorylation (Ser473) and p38 phosphorylation (Ser473) in splenic MRCs from three groups: MRC control, rVEGFA-supplemented and MSC-transwell coculture. (F) Representative western blot analysis of Erk1/2 phosphorylation (Thr202/Tyr204), Akt phosphorylation (Ser473) and p38 phosphorylation (Ser473) in splenic MRCs from three groups: MRC control, MSC-transwell coculture and MSC-transwell coculture with the selective VEGFR3 inhibitor SAR131675 (12 nM). All the experiments were performed with three independent biological replicates.

**Extended Data Fig. 19. Akt/Erk signaling in MRCs is the critical pathway mediating MSC-promoted MRC proliferation**

(A) Western blot analysis of Erk1/2 phosphorylation (Thr202/Tyr204) in splenic MRCs from three groups: MRC control, MSC-transwell coculture (control), and MSC-transwell coculture with the selective Erk1/2 inhibitor SCH772984 (4 nM). (B) Western blot analysis of Akt phosphorylation (Ser473) in splenic MRCs from three groups: MRC control, MSC-transwell coculture (control), and MSC-transwell coculture with the selective Akt inhibitor MK2206 (1 μM). (C) Western blot analysis of p38 phosphorylation (Ser473) in splenic MRCs from three groups: MRC control, MSC-transwell coculture (control), and MSC-transwell coculture with the selective p38 inhibitor Adezmapimod (5 μM). (D) Representative flow cytometry plots showing KI-67 expression in MRCs from the following groups: MRC control (treated with DMSO), MRC + MSC coculture, MRC + MSC coculture + the Erk1/2 inhibitor SCH772984, MRC + MSC coculture + the Akt inhibitor MK2206, and MRC + MSC coculture + the p38 inhibitor Adezmapimod. (E) Quantitative analysis of KI-67-positive MRCs in the groups described in (D). The data are presented as the means ± SEMs from 3 or more independent experiments. Statistical significance was determined via one-way ANOVA with multiple comparisons. *P < 0.05, **P < 0.01, ***P < 0.001, ****P < 0.0001; ns, not significant.
