# Supplementary material for: Mesenchymal stromal cells counteract with age-related immune decline and enhance vaccine efficacy by modulating endogenous splenic marginal reticular cells in elderly models
Source: Cell Mol Immunol. 2026 Jan 9;23(2):220–35. doi: 10.1038/s41423-025-01381-9 (PMC12858895; doi:10.1038/s41423-025-01381-9)
Supplement: Supplementary file 3 — Source Figure data [file 41423_2025_1381_MOESM3_ESM.pdf]

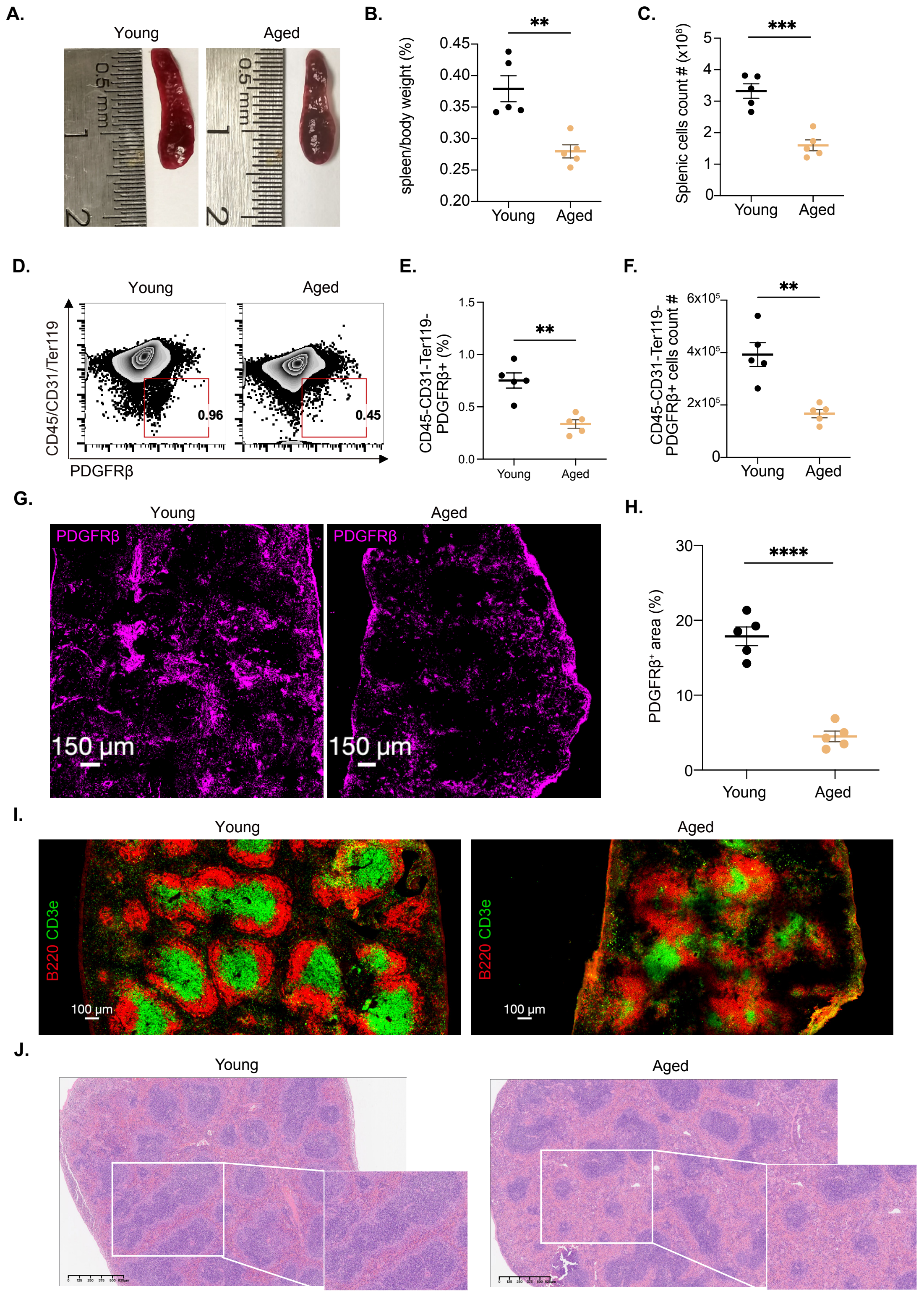

Extended Data Fig. 1. Diminished Splenic Stromal Cells and Architectural Defects in Aged Mice

(A) Representative gross observation of aged (>18-month-old) Balb/c spleen in the groups of Young (2-3 months) and Aged (>18-month-old) in mice. (B) Statistics

analysis of spleen weight/body weight ratio in the groups of Young and Aged, n=5 mice per group. (C) Statistics analysis of spleen cell counts in the groups of Young and Aged, n=5 mice per group. (D) Representative flow cytometry of splenic stromal cells proportion ($CD45^{-}CD31^{-}Ter119^{-}PDGFR\beta^{+}$) in the groups of Young and Aged. (E) Statistics analysis of splenic stromal cells proportion ($CD45^{-}CD31^{-}Ter119^{-}PDGFR\beta^{+}$) in the groups of Young and Aged, n=5 mice per group. (F) Statistics analysis of splenic stromal cells count in the groups of Young and Aged, n=5 mice per group. (G) Representative immunofluorescence staining of splenic stromal cells ($PDGFR\beta^{+}$, fuchsia) in the groups of Young and Aged. (H) Statistics analysis of splenic stromal cells area in the groups of Young and Aged, n=5 mice per group. (I) Representative immunofluorescence staining of splenic T lymphocytes ($CD3e^{+}$, green) and B lymphocytes ($B220^{+}$, red) in the groups of Young and Aged. (J) Representative hematein eosin staining of spleen in the groups of Young and Aged. Data represent mean ± SEM of 3 or more independent experiment. Statistical significance was determined using a two-tail unpaired t test. *$P < 0.05$, **$P < 0.01$, ***$P < 0.001$, ****$P < 0.0001$. ns, not significant.

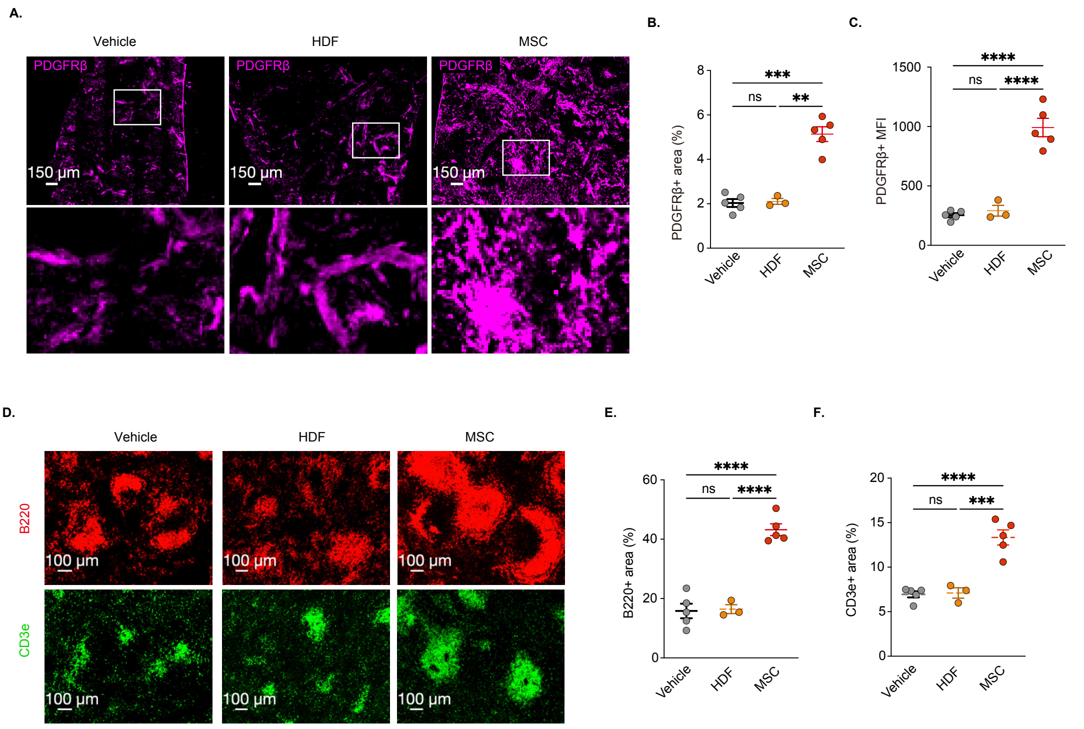

Extended Data Fig. 2. MSCs Promote Splenic Stromal Cell Architecture and Lymphocyte Expansion in Aged Mice

(A) Representative immunofluorescence staining of splenic stromal cells (PDGFRβ$^+$, fuchsia) in the groups of control (Vehicle), HDFs administration 28 days (HDF), and MSCs administration 28 days (MSC), scale bars: 150 μm. (B) Statistics analysis of splenic stromal cells area in the groups of Vehicle, HDF, MSC, n = 5 mice per group. (C) Statistics analysis of splenic stromal cells mean fluorescence intensity (MFI) in the groups of Vehicle, HDF, MSC, n = 5 mice per group. (D) Representative immunofluorescence staining of splenic T lymphocytes (CD3e$^+$, green) and B lymphocytes (B220$^+$, red) in the groups of Vehicle, HDF, MSC, scale bars: 100 μm. (E-F) Statistics analysis of B lymphocytes area and T lymphocytes area in the groups of Vehicle, MSC 1-4 weeks, n = 5 mice per group. Data represent mean ± SEM of 3 or more independent experiment. Statistical significance was determined using a one-way ANOVA with multiple comparison test. *$P < 0.05$, **$P < 0.01$, ***$P < 0.001$, ****$P < 0.0001$. ns, not significant.

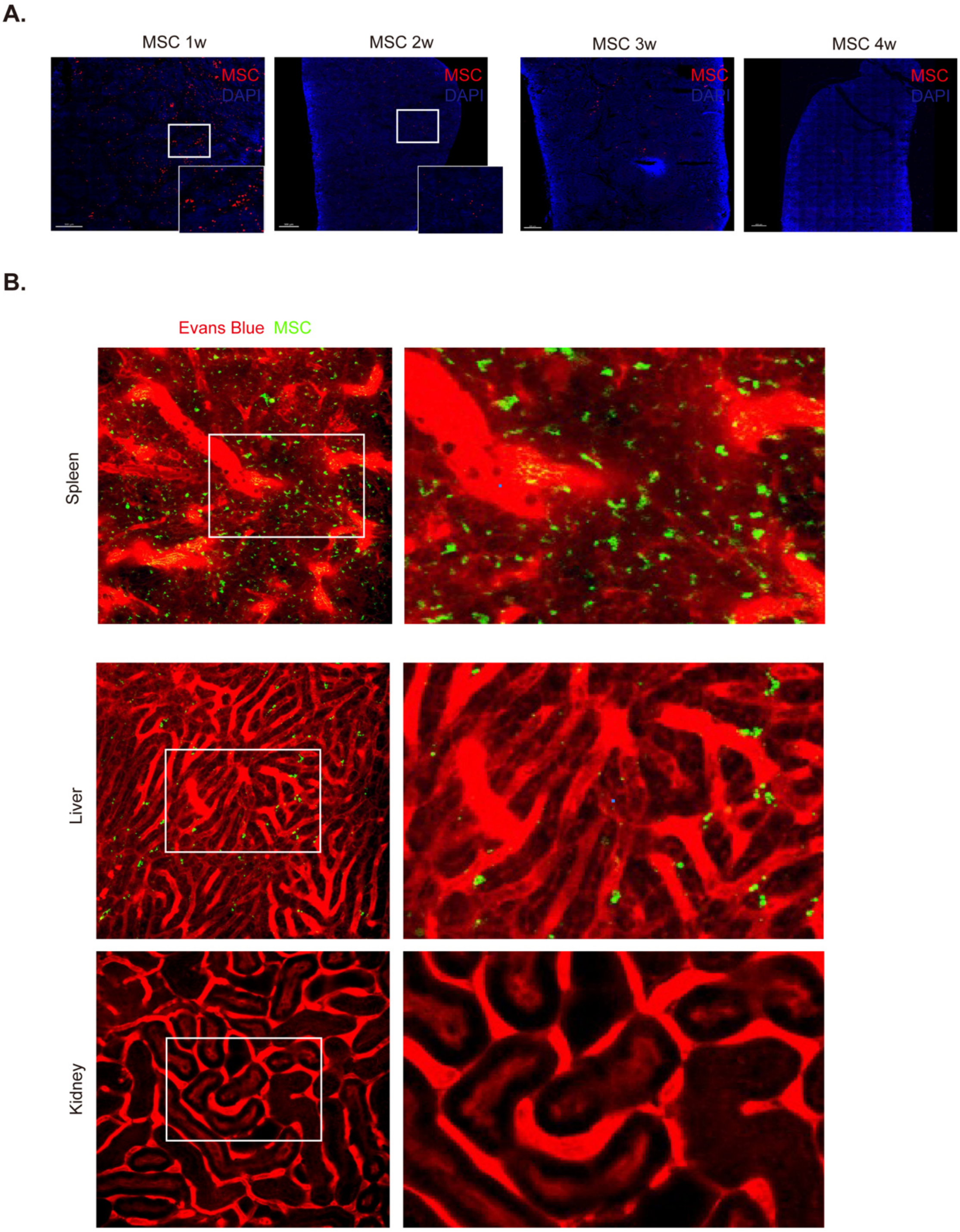

Extended Data Fig. 3. Residence of MSCs in the spleen.

(A) Representative immunofluorescence of MSCs (RFP^+) and DAPI (blue) in the groups of 1,2,3, and 4 weeks after MSCs infusion. (B) MSCs distributed in the spleen, liver and kidney in vivo.

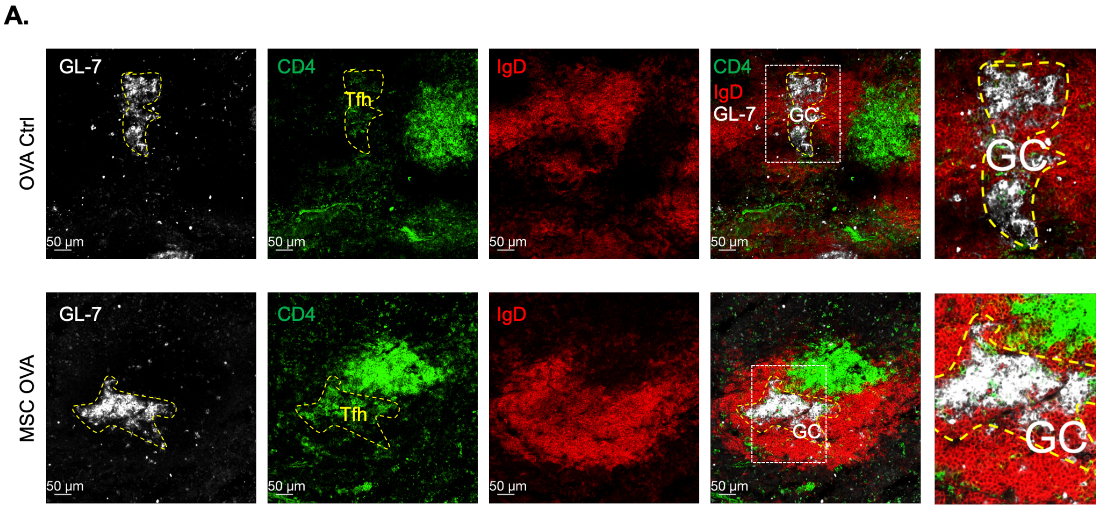

Extended Data Fig. 4. MSCs promote aged spleen germinal center formation after OVA immunization.

(A) Co-localization between Follicular B (FOB) cells clusters, T follicular helper (TFH) cells clusters and GC. Follicular B cells cluster (IgD^+, red), T follicular helper cells ($CD4^+$ in GC, green), Germinal center B cells (GL-7^+, white), scale bar: 50 μm.

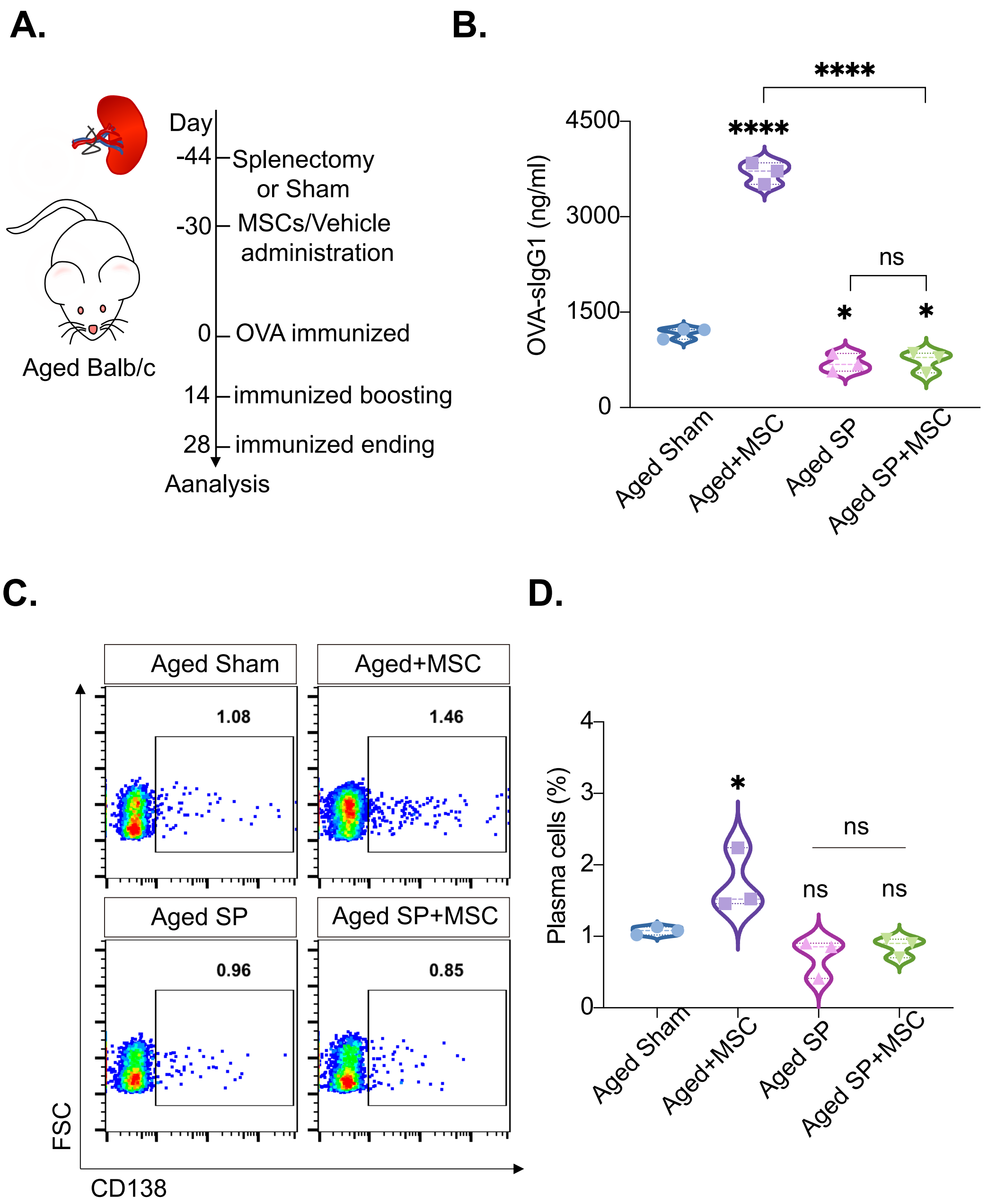

Extended Data Fig. 5. Spleen is an important target tissue for MSCs to enhance aged-related immune response.

(A) Schematic diagram of splenectomy mouse model, MSC infusion and immunization. (B) Concentrations of serum soluble ovalbumin-specific IgG1 antibodies after 28 days immunization in groups of aged sham, aged + MSC, aged splenectomy and aged splenectomy + MSC. (C) Representative flowcytometry of plasma cells (gated on B220^{+}, CD138^{+}) in groups of aged sham, aged + MSC, aged splenectomy and aged splenectomy + MSC. (D) Statistics analysis of plasma cells in groups of aged sham, aged + MSC, aged splenectomy and aged splenectomy + MSC. Data represent mean ±

SEM of 3 or more independent experiment. Statistical significance was determined using a one-way ANOVA with multiple comparison test. *P < 0.05, **P < 0.01, ***P < 0.001, ****P < 0.0001. ns, not significant.

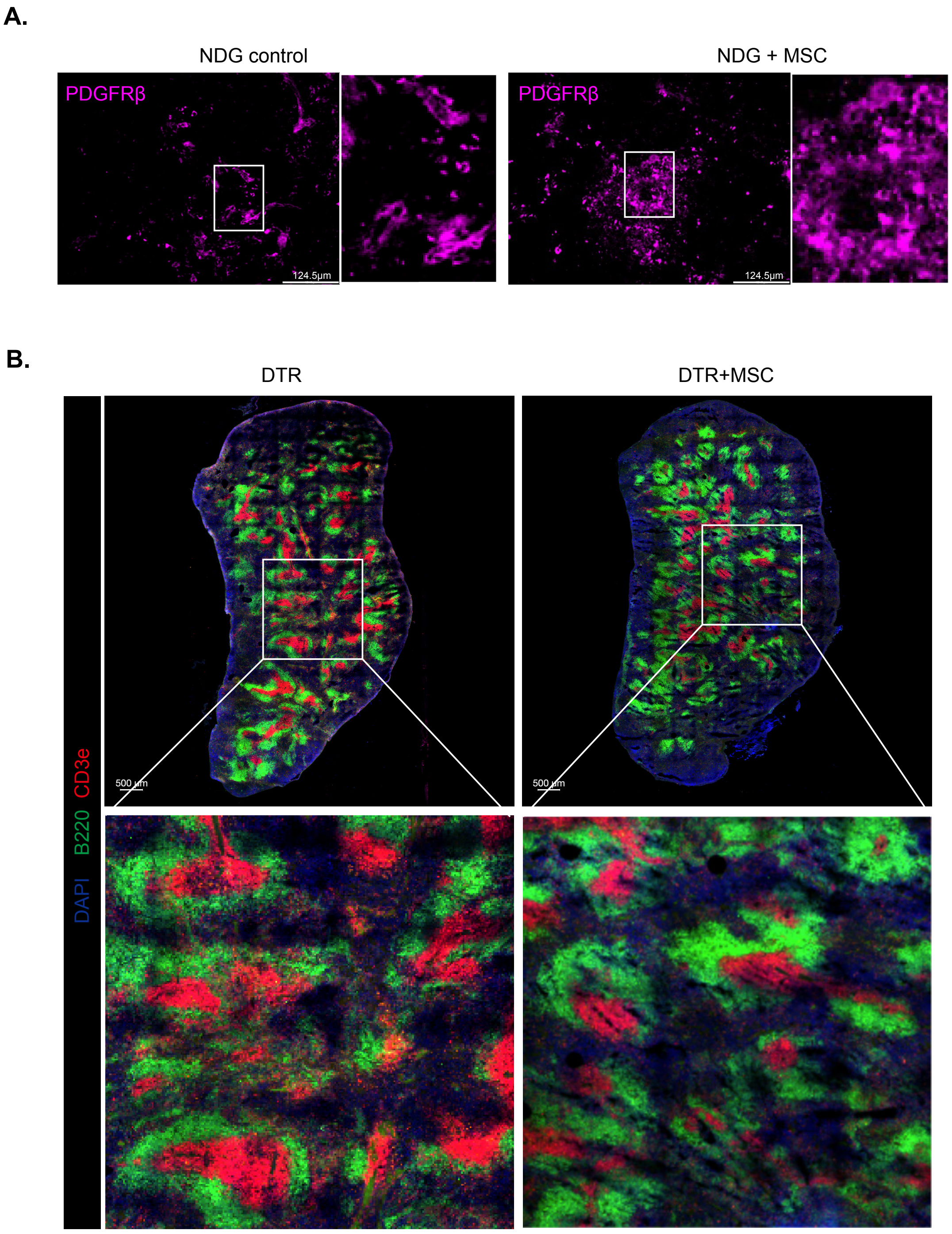

Extended Data Fig. 6. MSCs restore spleen microarchitectures mainly by modulating stromal cells

(A) Representative immunofluorescence staining of splenic stromal cells (PDGFRβ$^+$, fuchsia) in the groups of NDG control and MSCs administration 28 days (NDG+MSC), scale bars: 124.5 μm. (B) Representative immunofluorescence staining of DAPI (Blue), splenic T lymphocytes (CD3e$^+$, red) and B lymphocytes (B220$^+$, green) in the groups

of splenic stromal cells depletion control (DTR) and MSCs administration (DTR+MSC), scale bars: 500 μm.

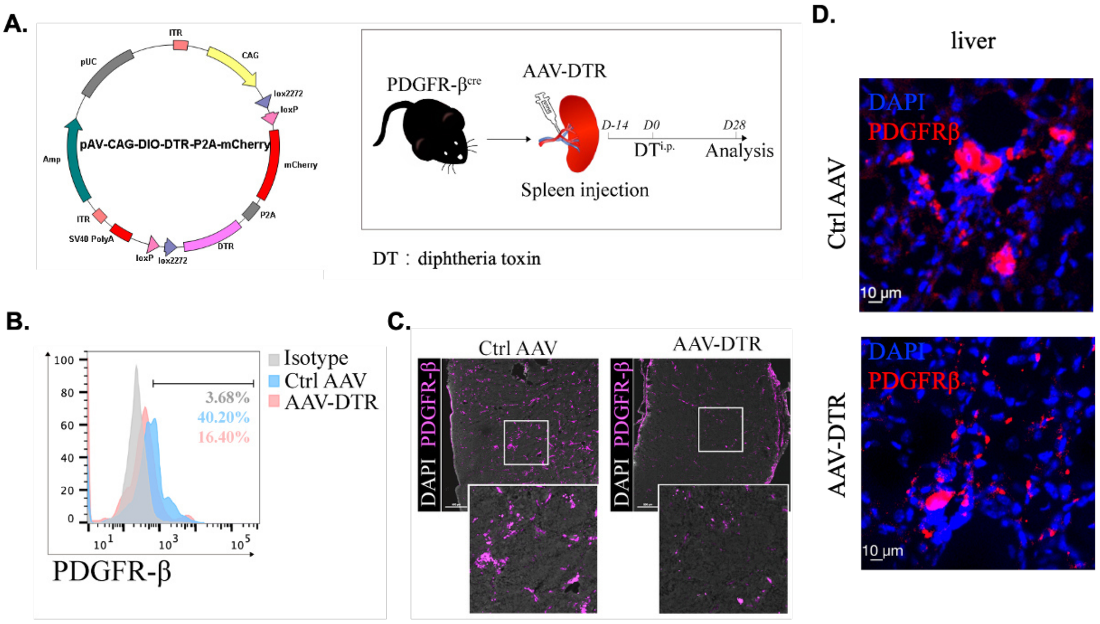

Extended Data Fig. 7. PDGFRβ-cre × AAV-iDTR system established to selectively deplete splenic stromal populations.

(A) adeno-associated virus (AAV) carrying a Cre-dependent construct (PAV-CAG-DIO-DTR-P2A-mCherry) system to selectively deplete splenic stromal cells. (B) The splenic stromal cell population was detected by flow cytometry in the group of Ctrl AAV and AAV-DTR. (C) Representative immunofluorescence images were used to detect PDGFRβ$^+$ cells of spleens in the group of Ctrl AAV and AAV-DTR, scale bar: 500 μm. (d) Representative immunofluorescence images were used to detect PDGFRβ$^+$ cells of livers in the group of Ctrl AAV and AAV-DTR, scale bar: 10 μm.

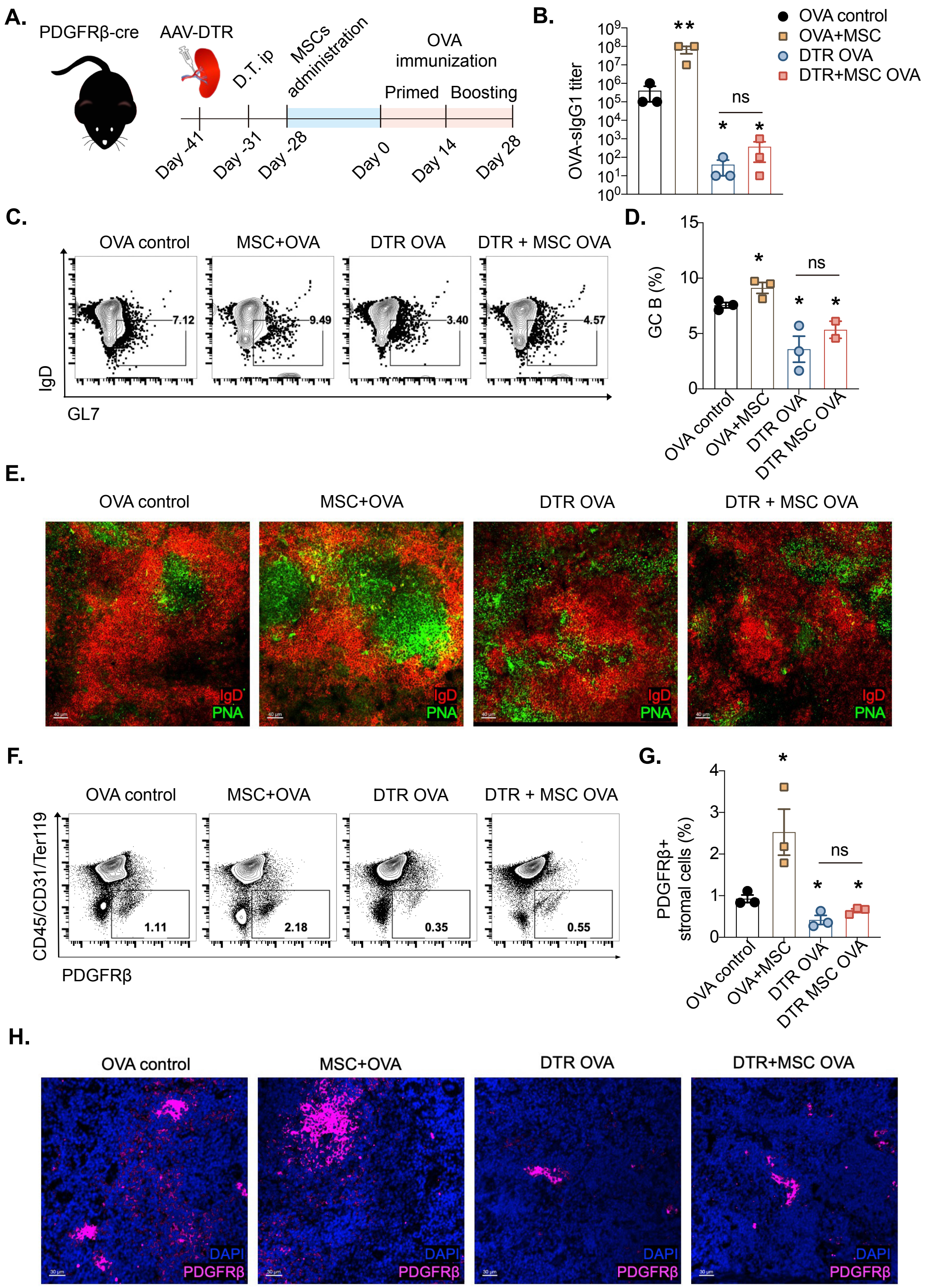

Extended Data Fig. 8. MSCs could not produce the level of specific antibodies in mice when specific spleen stromal cells deficiency

(A) AAV-DTR injection specifically cleared splenic stromal cells, followed by MSC infusion and OVA immunization in the group of control AAV (OVA control), control

AAV and MSCs administration (MSC+OVA), AAV-DTR control (DTR OVA) and AAV-DTR and MSCs administration (DTR+MSC OVA). (B) Titer of serum total IgG1 antibodies after 28 days immunization in the group of OVA control, MSC+OVA, DTR OVA and DTR+MSC OVA. (C) Representative flow cytometry of splenic germinal center B cell populations ($B220^{+}IgD^{-}GL7^{+}$) in the group of OVA control, MSC+OVA, DTR OVA and DTR+MSC OVA. (D) Statistics analysis of splenic germinal center B cells proportion in the group of OVA control, MSC+OVA, DTR OVA and DTR+MSC OVA, n=3 mice per group. (E) Representative immunofluorescence staining of follicular B lymphocytes (IgD^{+}, red) and germinal center (PNA^{+}, green) in the group of OVA control, MSC+OVA, DTR OVA and DTR+MSC OVA. (F) Representative flow cytometry of splenic stromal cells ($CD45^{-}CD31^{-}Ter119^{-}PDGFR\beta^{+}$) in the group of OVA control, MSC+OVA, DTR OVA and DTR+MSC OVA. (G) Statistics analysis of splenic stromal cells proportion in the group of OVA control, MSC+OVA, DTR OVA and DTR+MSC OVA, n=3 mice per group. (H) Representative immunofluorescence staining of splenic stromal cells ($PDGFR\beta^{+}$, fuchsia) in the group of OVA control, MSC+OVA, DTR OVA and DTR+MSC OVA. Data represent mean ± SEM of 3 or more independent experiment. Statistical significance was determined using a one-way ANOVA with multiple comparison test. *$P < 0.05$, **$P < 0.01$, ***$P < 0.001$, ****$P < 0.0001$. ns, not significant.

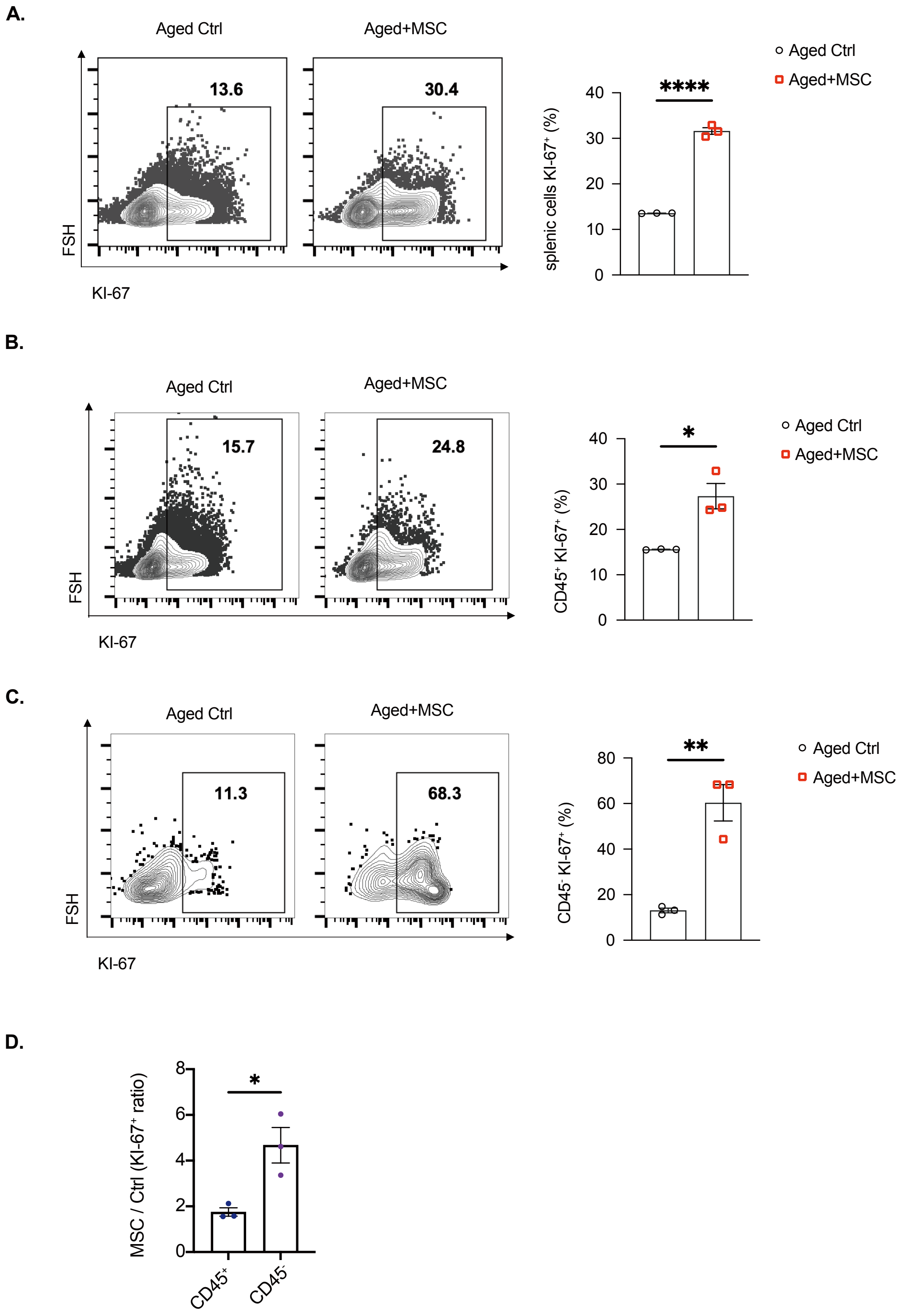

Extended Data Fig. 9. MSCs Promotion of Splenic Cell Proliferation in Aged mice, Particularly CD45$^-$ Cells

(A) Flow cytometry analysis of splenic cells proliferation proportion（KI-67$^+$) in the groups of vehicle (Aged Ctrl) or MSCs (Aged + MSC)administration after 3 days, n=3

mice per group. (B) Flow cytometry analysis of splenic immune cells proliferation proportion （$CD45^{+}KI\text{-}67^{+}$) in the groups of vehicle (Aged Ctrl) or MSCs (Aged + MSC)administration after 3 days, n=3 mice per group. (C) Flow cytometry analysis of splenic structural cells proliferation proportion （$CD45^{-}KI\text{-}67^{+}$) in the groups of vehicle (Aged Ctrl) or MSCs (Aged + MSC) administration after 3 days, n=3 mice per group. (D) Statistics analysis of splenic cells proliferation proportion ration between MSCs administration to Aged Ctrl in the groups of $CD45^{-}$ and $CD45^{+}$ splenic cells, n=3 mice per group. Data represent mean ± SEM of 3 or more independent experiment. Statistical significance was determined using a two-tail unpaired t test. *$P < 0.05$, **$P < 0.01$, ***$P < 0.001$, ****$P < 0.0001$. ns, not significant.

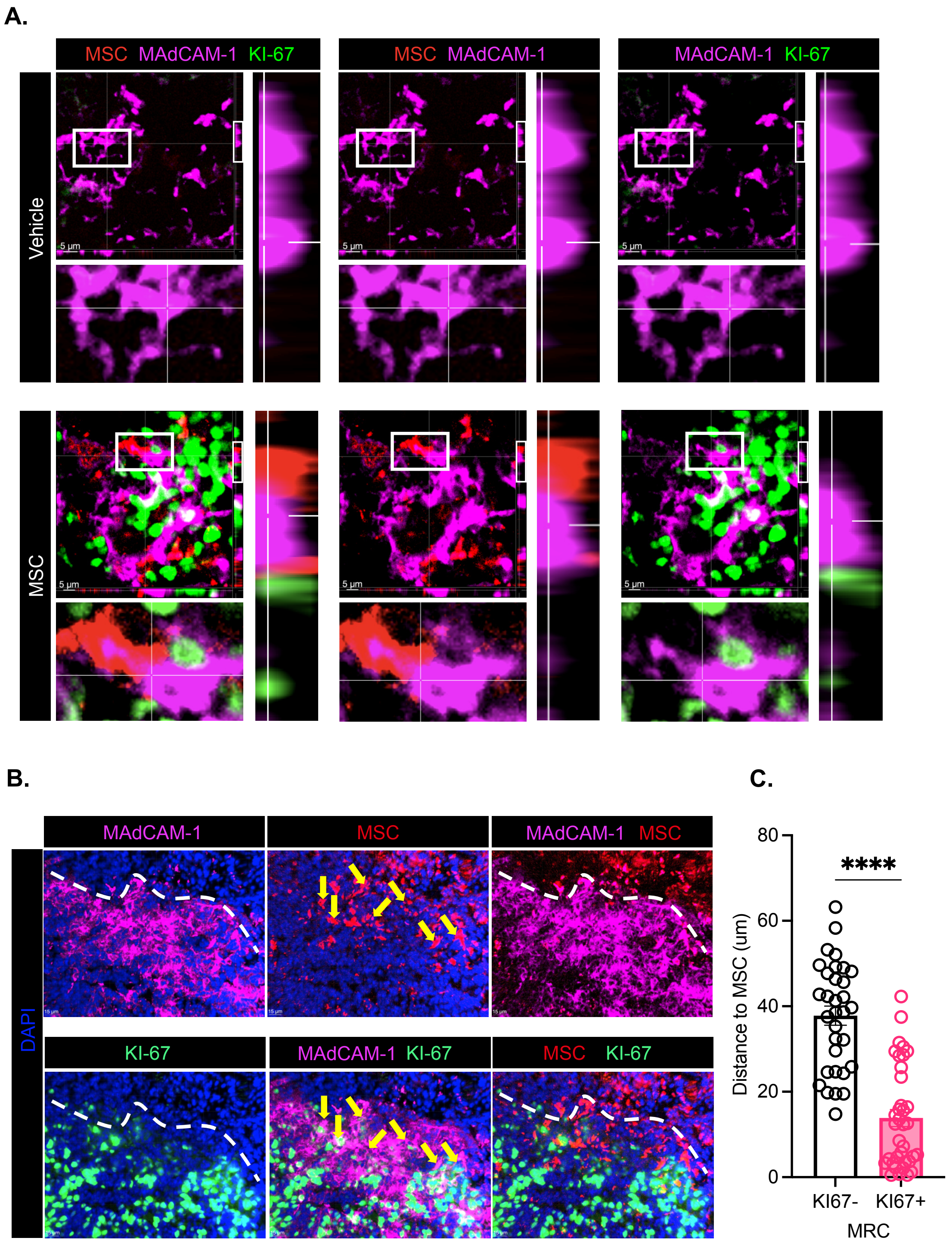

Extended Data Fig. 10. Spatial proximity between MSCs and MRCs promotes KI-67 expression in MRCs

(A) Representative immunofluorescence staining of KI-67 (green) expression in splenic MRCs (MAdCAM-1^{+}, fuchsia) in the group of the vehicle and MSCs administration.

(B) Representative fluorescence staining of MRCs (KI-67^{+}; KI-67^{-}) and their distance from MSCs. (C) Statistics analysis of MRCs (KI-67^{+}; KI-67^{-}) and their distance from MSCs. Data represent mean ± SEM of 3 or more independent experiment. Statistical significance was determined using a two-tail unpaired t test. *P < 0.05, **P < 0.01, ***P < 0.001, ****P < 0.0001. ns, not significant.

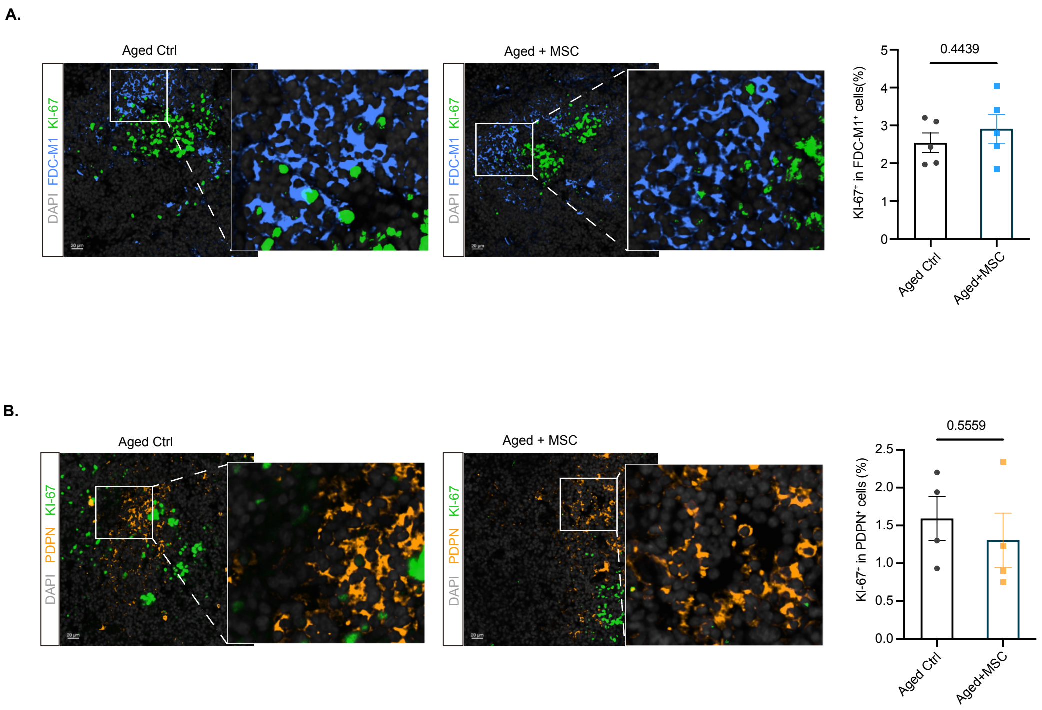

Extended Data Fig. 11. Limited Impact of MSCs on the Proliferation of Splenic FDC and FRC Cells in Aged Mice

(A) Representative immunofluorescence staining of splenic follicular dendritic cells proliferation (FDC-M1$^+$, blue, KI-67$^+$, green and DAPI, grey) and analysis in the groups of vehicles (Aged Ctrl) or MSCs (Aged + MSC) administration after 3 days, n=5 mice per group. (B) Representative immunofluorescence staining of splenic follicular reticular cells proliferation (PDPN$^+$, orange, KI-67, green and DAPI, grey) and analysis in the groups of vehicles (Aged Ctrl) or MSCs (Aged + MSC) administration after 3 days, n=5 mice per group. Data represent mean ± SEM of 3 or more independent experiment. Statistical significance was determined using a two-tail unpaired t test. *P < 0.05, **P < 0.01, ***P < 0.001, ****P < 0.0001. ns, not significant.

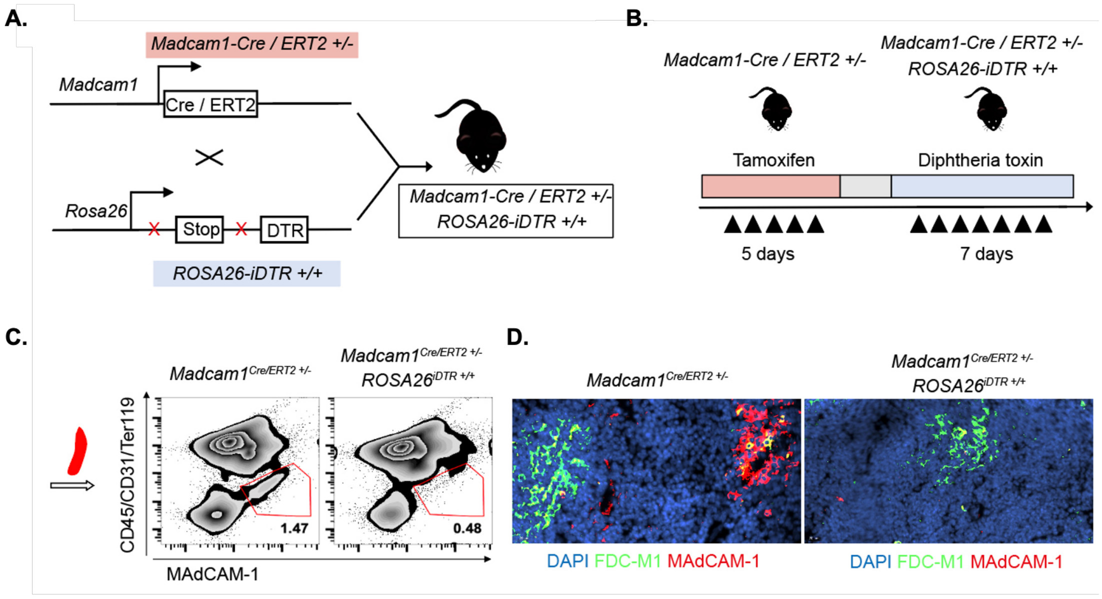

Extended Data Fig. 12. Limited Impact of MSCs on the Proliferation of Splenic FDC and FRC Cells in Aged Mice.

(A) Construction of genetic mice with tamoxifen induced diphtheria toxin conditional ablation of MRC (*Madcam-1 cre/ERT2* x *ROSA26 iDTR*). (B) Tamoxifen induced diphtheria toxin conditional ablation in *Madcam-1 cre/ERT2* x *ROSA26 iDTR* mice and *Madcam-1 cre/ERT2* mice as non-ablation control. (C) The ablation effect of MRCs in spleen was detected by flow cytometry ($CD45^-CD31^-Ter119^-MAdCAM\text{-}1^+$). (D) Representative immunofluorescence results were used to detect MRC ($MAdCAM\text{-}1^+$, red) and FDC ($FDC\text{-}M1^+$, green) of spleens in the group of *Madcam-1 cre/ERT2* and *Madcam-1 cre/ERT2* x *ROSA26 iDTR.*

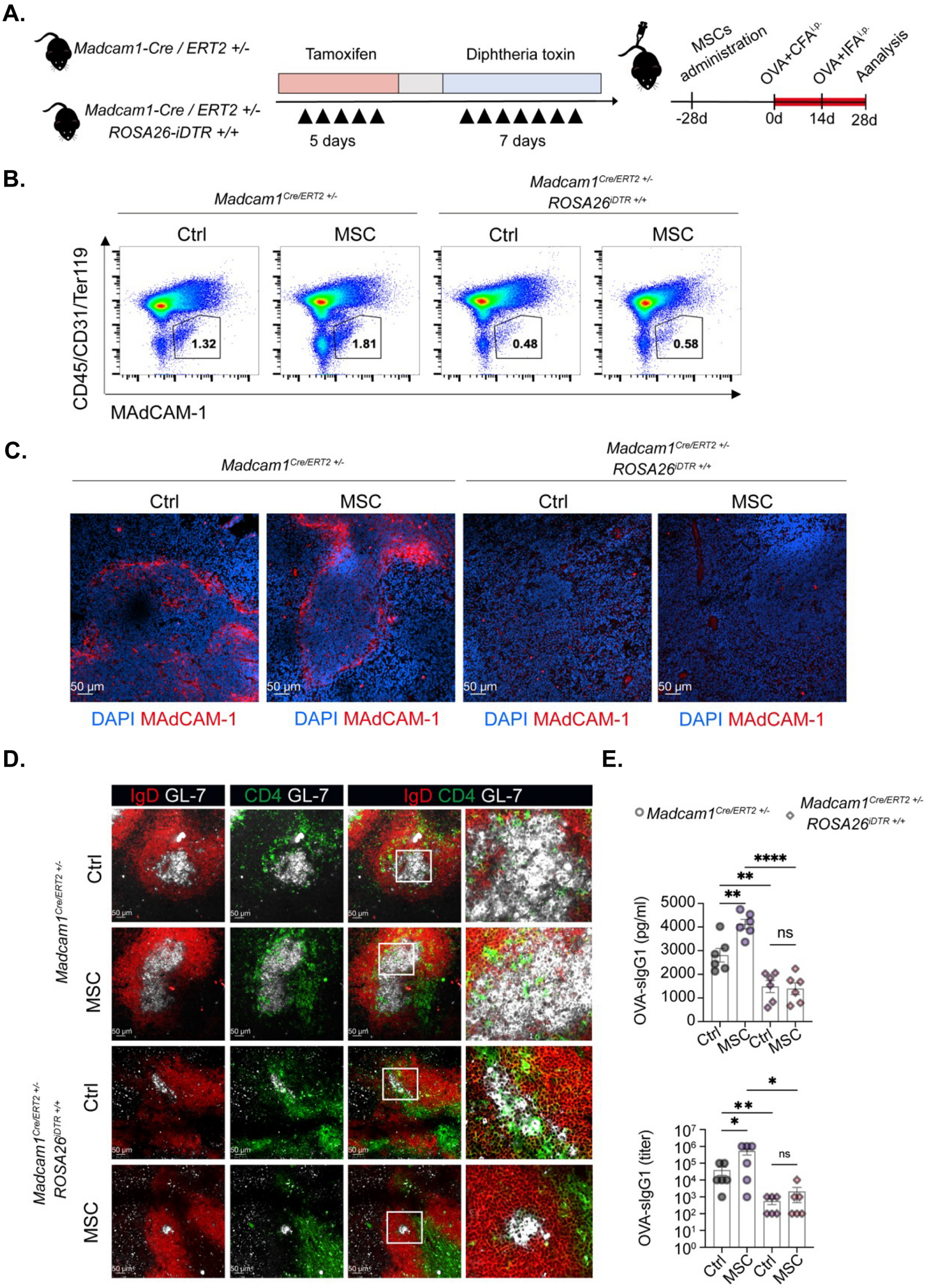

Extended Data Fig. 13. Following MRC deficiency, the ability of MSCs to promote splenic germinal center formation and enhance OVA-specific antibody production was abolished.

(A) OVA immune procedure after MRCs ablation. (B) The ablation effect of MRCs in

spleen was detected by flow cytometry (CD45$^-$CD31$^-$Ter119$^-$MAdCAM-1$^+$). (C) Representative immunofluorescence results were used to detect MRC (MAdCAM-1$^+$, red) of spleens (scale bar: 50um). (D) Representative immunofluorescence results were used to detect GC (GL-7$^+$, white), FOB (IgD$^+$, red) and Tfh (CD4$^+$ co-localized in GC, green), scale bar: 50um. (E) Concentration and titers of serum soluble ovalbumin-specific IgG1 antibodies after 28 days immunization.

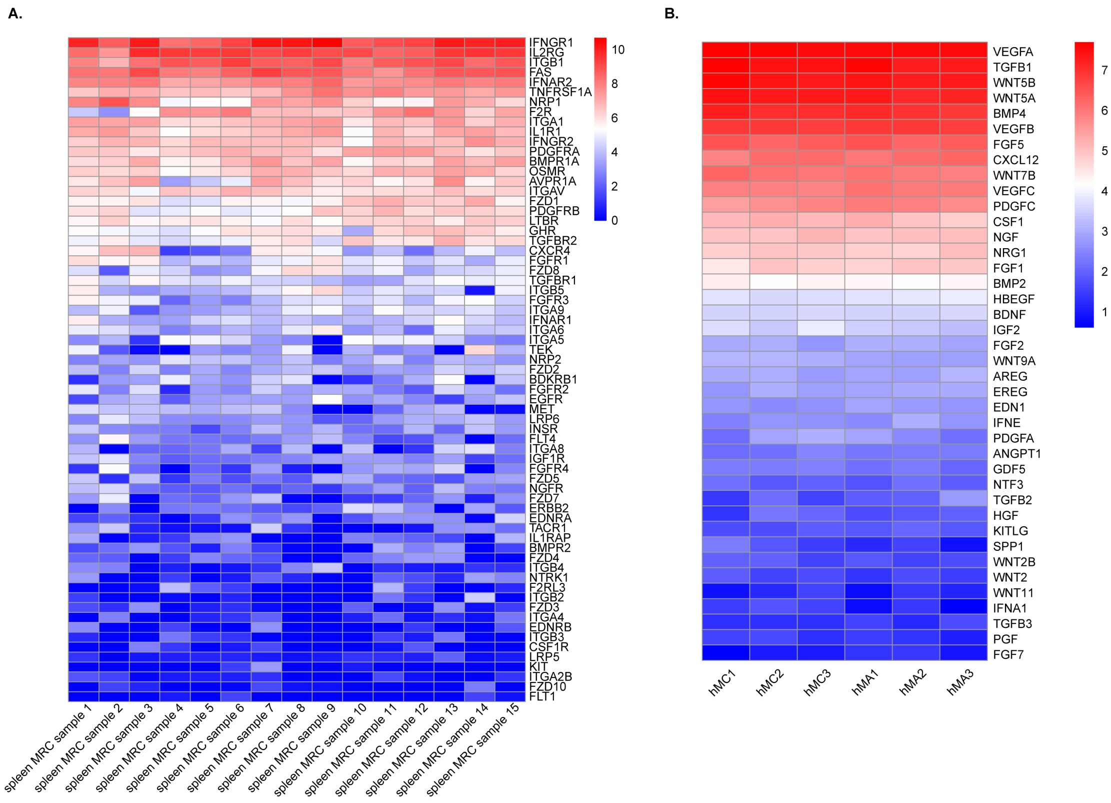

Extended Data Fig. 14. VEGFA is a Potential Functional Molecule of MSCs

(A) The top 67 receptor molecules associated with cell proliferation in spleen MRCs and ranked based on their expression levels. (B) Ligand molecules corresponding to (A) were screened and ranked using bulk RNA sequencing data from MSCs between naïve in vitro and in aged spleen.

Exten

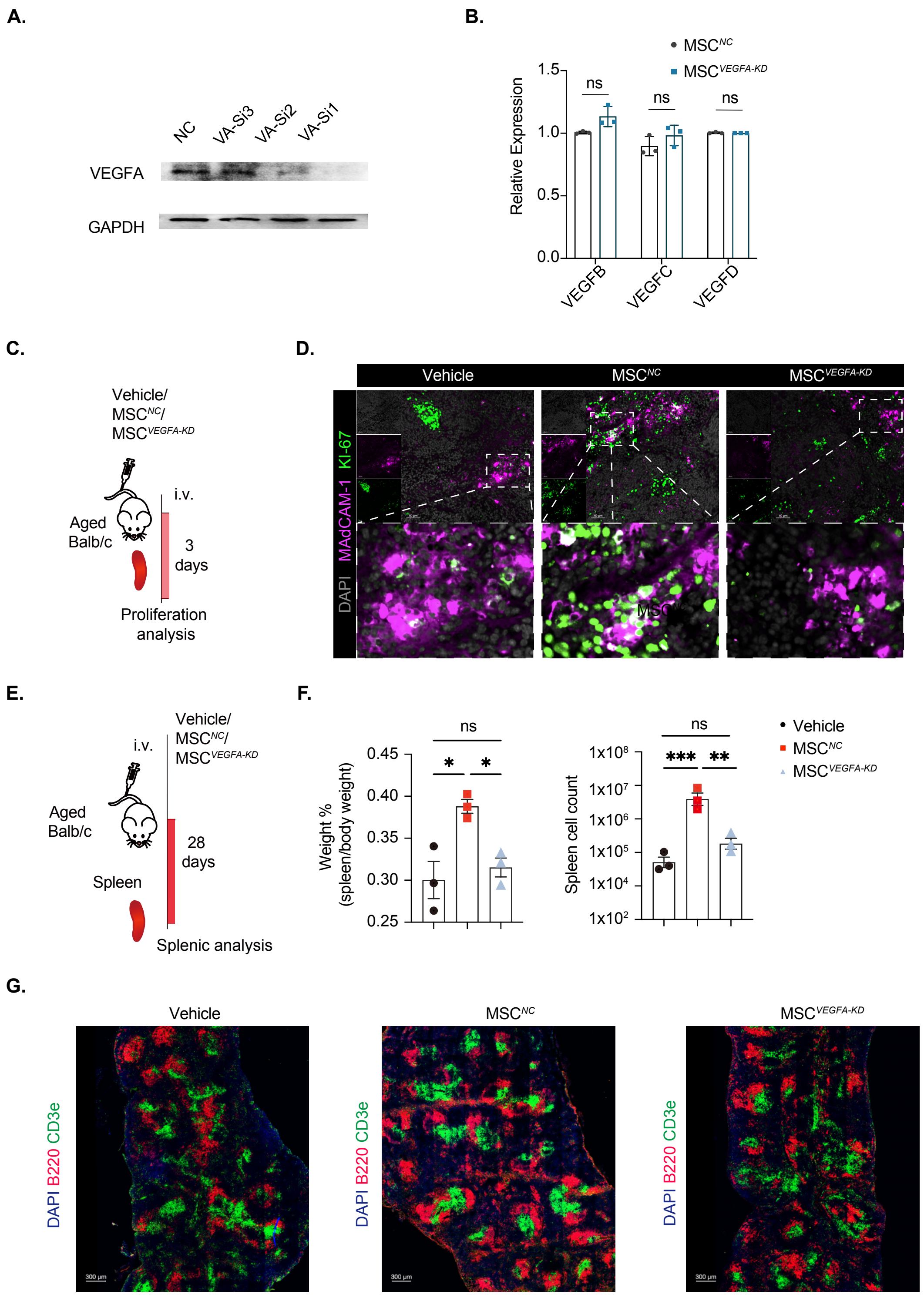

Extended Data Fig. 15. Impaired Function of MSCs in Promoting Splenic MRC Proliferation and Spleen Stromal and lymphocytes Architecture Recovery in Aged

Mice Due to Defective Expression of VEGFA

(A) VEGFA protein expression detection through Western blot in the groups of MSC^{NC} and $MSC^{VEGFA\text{-}KD1}$, $MSC^{VEGFA\text{-}KD2}$, $MSC^{VEGFA\text{-}KD3}$. (B) Relative VEGFB, VEGFC and VEGFD mRNA expression detection through RT-PCR validation in the groups of MSC^{NC} and $MSC^{VEGFA\text{-}KD}$, n=3. (C) Vehicles (Vehicle), non-sense control MSCs administration (MSC^{NC}) or VEGFA knock-down MSCs administration ($MSC^{VEGFA\text{-}KD}$) were administrated into aged balb/c mice (>18-month-old) and analyzed 3 days later. (D) Representative immunofluorescence staining of splenic MRC ($MAdCAM\text{-}1^{+}$, fuchsia), KI-67(green) and DAPI (gray) in the groups of Vehicle, MSC^{NC} and $MSC^{VEGFA\text{-}KD}$, scale bars: 40 μm. (E) Vehicles (Vehicle), non-sense control MSCs administration (MSC^{NC}) or VEGFA knock-down MSCs administration ($MSC^{VEGFA\text{-}KD}$) were administrated into aged balb/c mice (>18-month-old) and analyzed 30 days later. (F) Statistics analysis of spleen weight/body weight ratio and splenic cells count in the groups of Vehicle, MSC^{NC} and $MSC^{VEGFA\text{-}KD}$. (G) Representative immunofluorescence staining of splenic T lymphocytes ($CD3e^{+}$, green) and B lymphocytes ($B220^{+}$, red) in the groups of Vehicle, MSC^{NC} and $MSC^{VEGFA\text{-}KD}$, scale bars: 300 μm. Data represent mean ± SEM of 3 or more independent experiment. Statistical significance was determined using a one-way ANOVA with multiple comparison test. *P < 0.05, **P < 0.01, ***P < 0.001, ****P < 0.0001. ns, not significant.

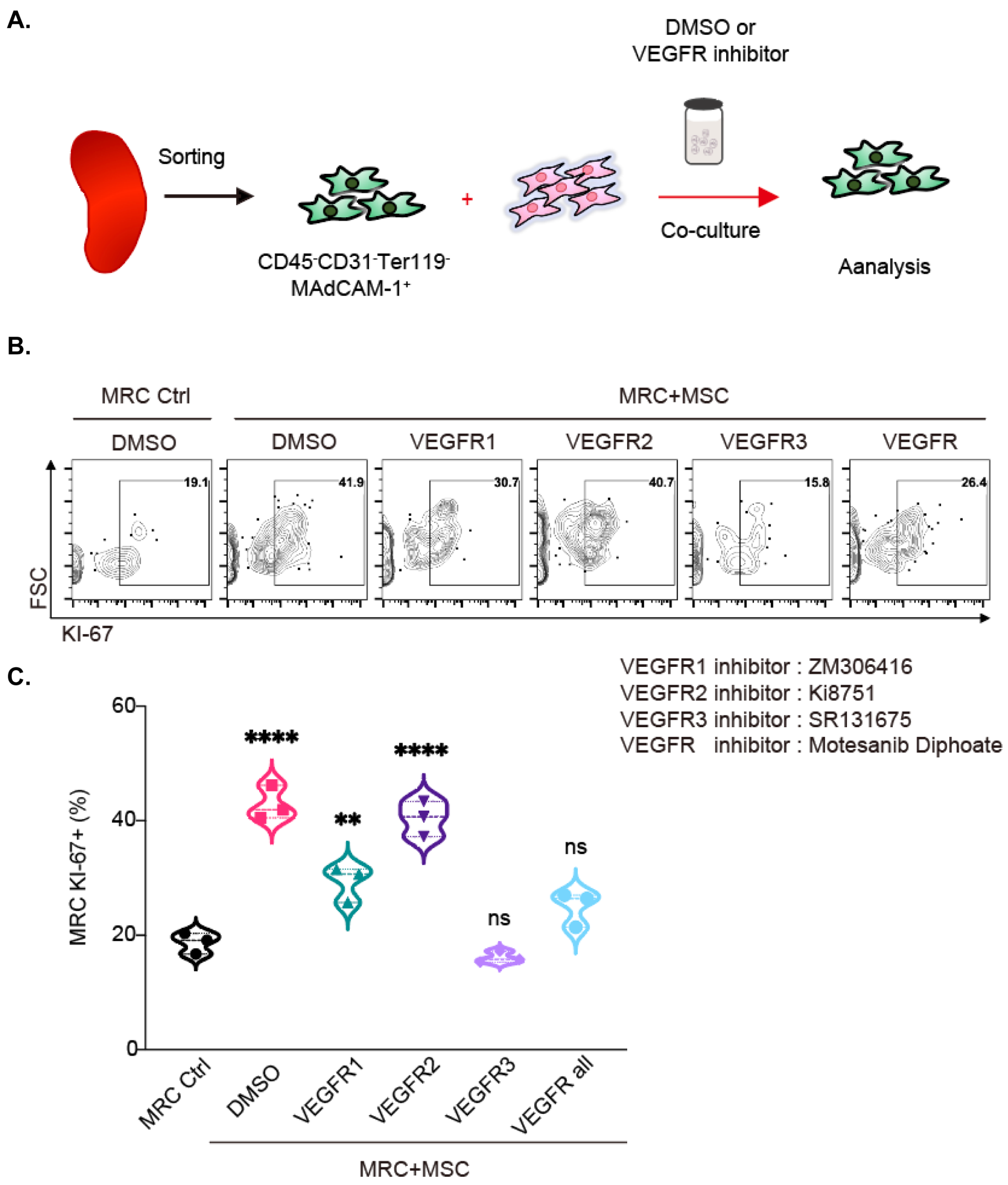

Extended Data Fig. 16. VEGFR3 blockade significantly reversed MSC-mediated MRCs proliferation.

(A) Schematic diagram of sorting splenic CD45$^-$CD31$^-$Ter119$^-$MadCAM-1$^+$ cells. (B) Representative plots of KI-67 expression by MRC in the groups of MRC control, MRC + MSC, MRC+MSC+VEGFR1 inhibitor, MRC+MSC+VEGFR2 inhibitor, MRC+MSC+VEGFR3 inhibitor and MRC+MSC+VEGFR inhibitor. (C) Analysis of KI-67 expression by MRC in the groups of MRC control, MRC + MSC, MRC+MSC+VEGFR1 inhibitor, MRC+MSC+VEGFR2 inhibitor,

MRC+MSC+VEGFR3 inhibitor and MRC+MSC+VEGFR inhibitor. Data represent mean ± SEM of 3 or more independent experiment. Statistical significance was determined using a one-way ANOVA with multiple comparison test. *P < 0.05, **P < 0.01, ***P < 0.001, ****P < 0.0001. ns, not significant.

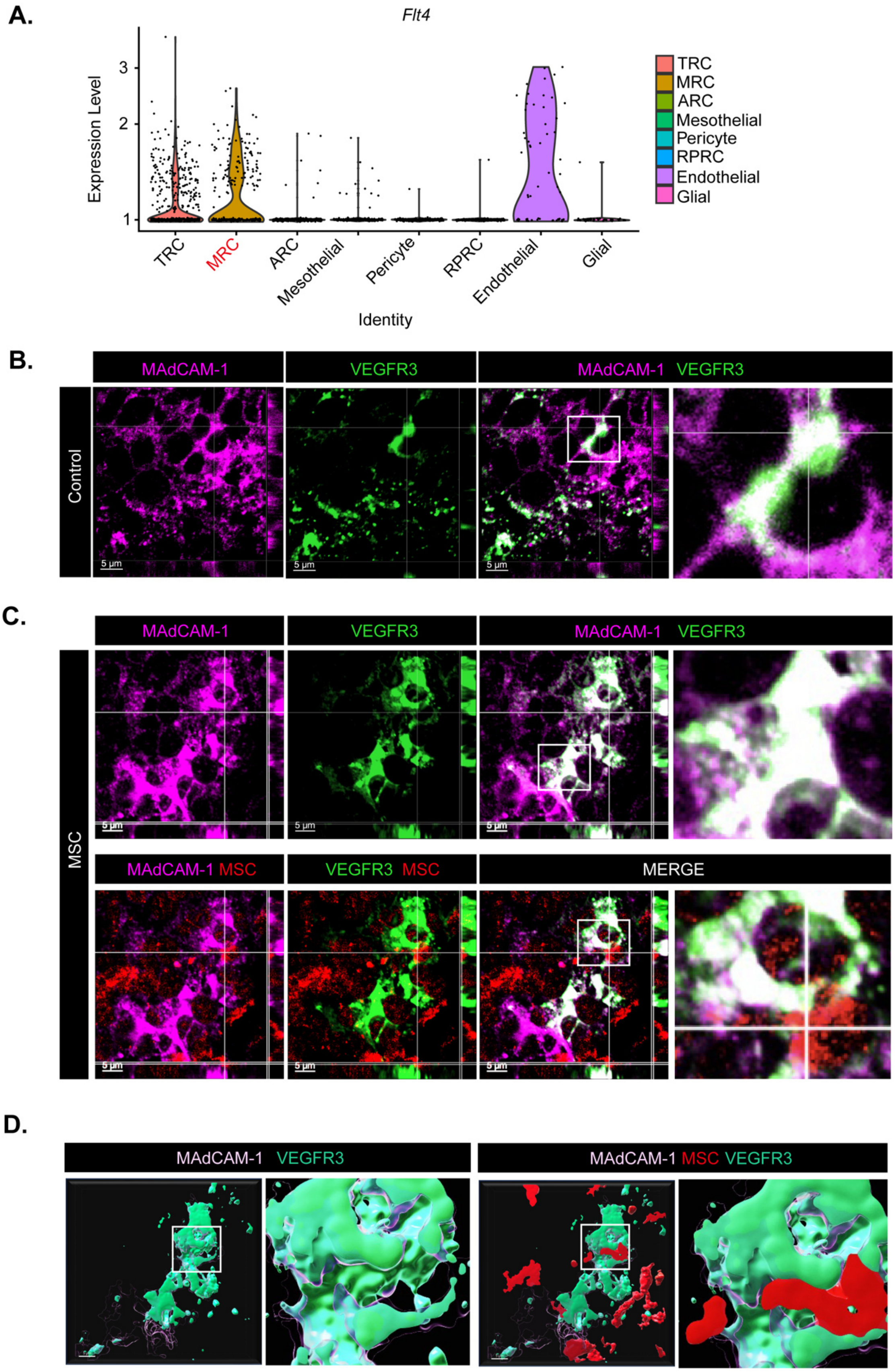

Extended Data Fig. 17. Splenic MRCs express VEGFR3

(A) Violin plots of FIt4 mRNA expression in spleen stromal subsets, data from

GSE274926. (B-C) Representative fluorescence staining of VEGFR3 (green) expression by MRC (MAdCAM-1^{+}, fuchsia) in the groups of Control and MSC (red) administration. (D) Representative 3D modeling diagram illustrating the spatial distribution of VEGFR3^{+} (green) MRCs (MAdCAM-1^{+}, fuchsia) in close proximity to MSCs (red) via fluorescence staining.

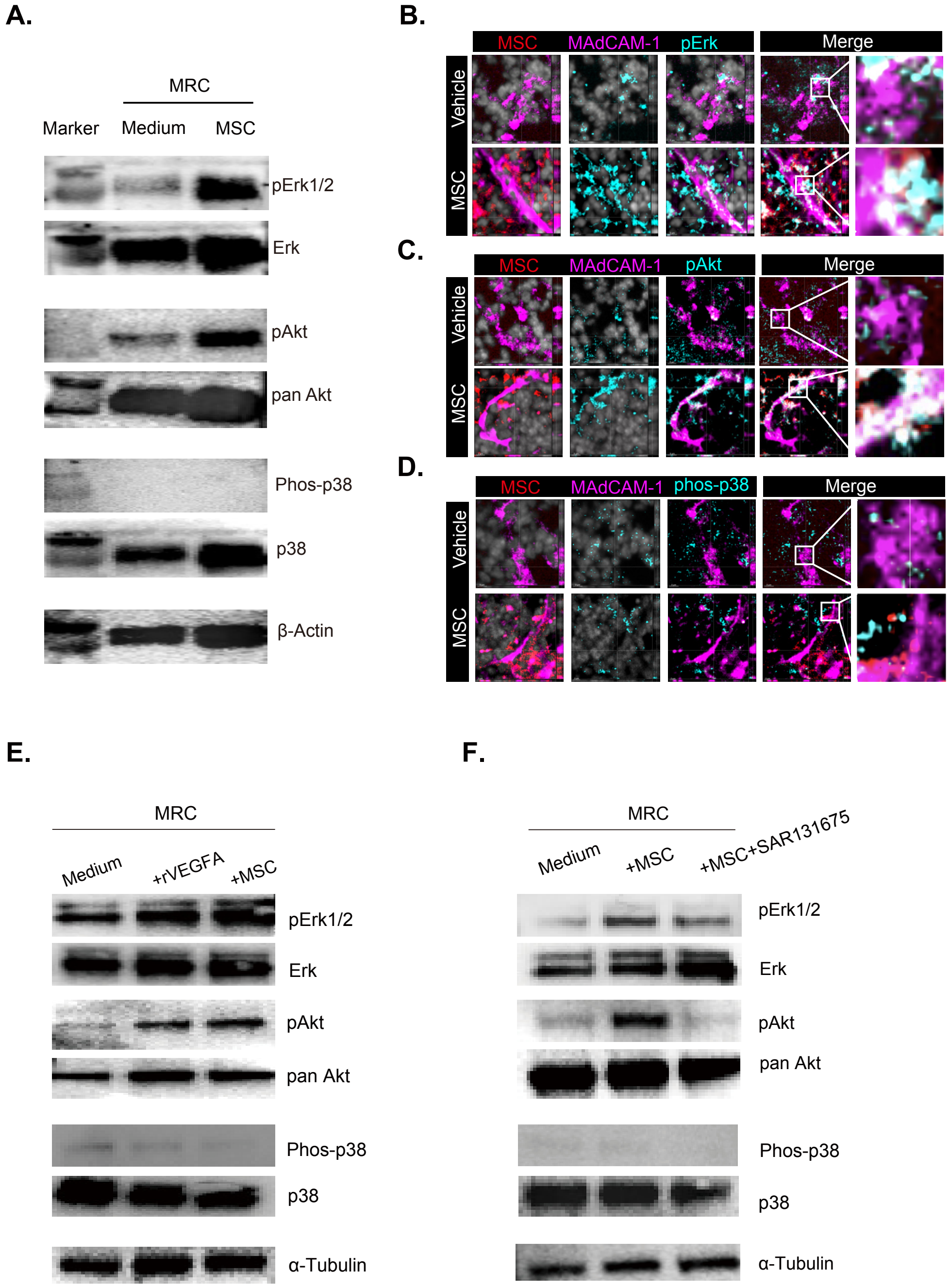

Extended Data Fig. 18. MSCs promote the phosphorylation of Akt/Erk in splenic MRCs through VEGFA-VEGFR3 axis

(A) Representative western blot analysis of Erk1/2 phosphorylation (Thr202/Tyr204), Akt phosphorylation (Ser473) and p38 phosphorylation (Ser473) in splenic MRCs from two groups: MRC control and MSC-transwell co-culture. (B) Representative immunofluorescence staining showing Erk1/2 phosphorylation (Thr202/Tyr204, turquoise) in splenic MRCs (MAdCAM-1^{+}, fuchsia) from mice treated with vehicle or

MSCs. (C) Representative immunofluorescence staining showing Akt phosphorylation (Ser473, turquoise) in splenic MRCs (MAdCAM-1$^+$, fuchsia) from mice treated with vehicle or MSCs. (D) Representative immunofluorescence staining showing p38 phosphorylation (Ser473, turquoise) in splenic MRCs (MAdCAM-1$^+$, fuchsia) from mice treated with vehicle or MSCs. (E) Representative western blot analysis of Erk1/2 phosphorylation (Thr202/Tyr204), Akt phosphorylation (Ser473) and p38 phosphorylation (Ser473) in splenic MRCs from three groups: MRC control, rVEGFA supplied and MSC-transwell co-culture. (F) Representative western blot analysis of Erk1/2 phosphorylation (Thr202/Tyr204), Akt phosphorylation (Ser473) and p38 phosphorylation (Ser473) in splenic MRCs from three groups: MRC control, MSC-transwell co-culture and MSC-transwell co-culture with the selective VEGFR3 inhibitor SAR131675 (12 nM). All experiments were performed with three independent biological replicates.

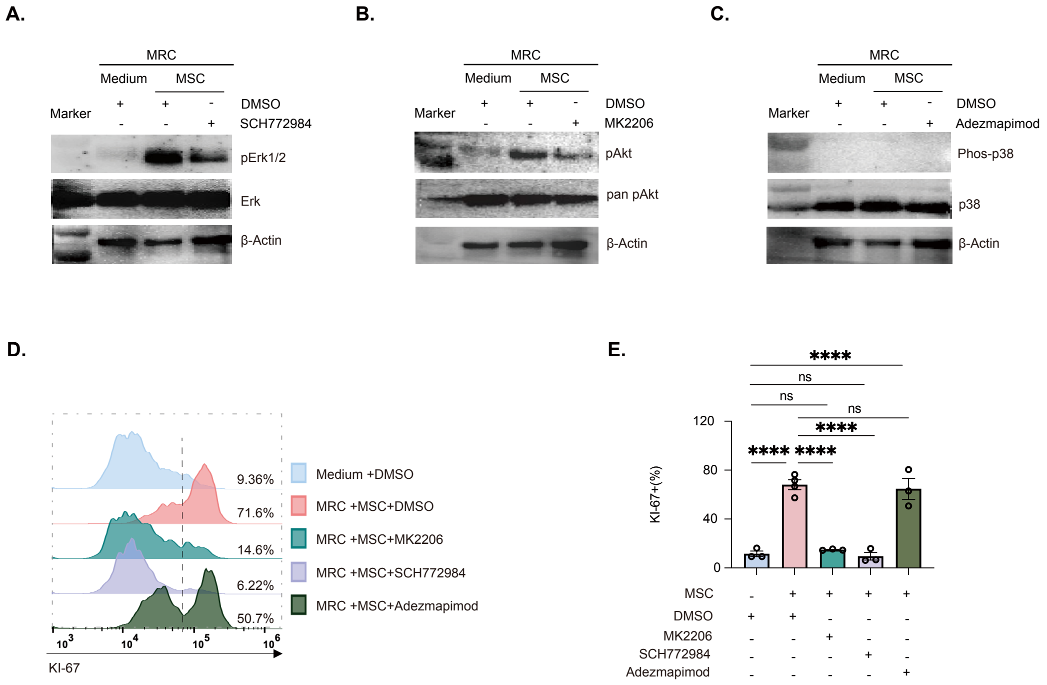

Extended Data Fig. 19. Akt/Erk signaling in MRCs is the critical pathway mediating MSC-promoted MRC proliferation

(A) Western blot analysis of Erk1/2 phosphorylation (Thr202/Tyr204) in splenic MRCs from three groups: MRC control, MSC-transwell co-culture (control), and MSC-transwell co-culture with the selective Erk1/2 inhibitor SCH772984 (4 nM). (B) Western blot analysis of Akt phosphorylation (Ser473) in splenic MRCs from three groups: MRC control, MSC-transwell co-culture (control), and MSC-transwell co-culture with the selective Akt inhibitor MK2206 (1 µM). (C) Western blot analysis of p38 phosphorylation (Ser473) in splenic MRCs from three groups: MRC control, MSC-transwell co-culture (control), and MSC-transwell co-culture with the selective p38 inhibitor Adezmapimod (5 µM). All experiments were performed with three independent biological replicates. (D) Representative flow cytometry plots showing KI-67 expression in MRCs from the following groups: MRC control (treated with DMSO), MRC + MSC co-culture, MRC + MSC co-culture + Erk1/2 inhibitor SCH772984, MRC + MSC co-culture + Akt inhibitor MK2206, and MRC + MSC co-culture + p38 inhibitor Adezmapimod. (E) Quantitative analysis of KI-67-positive MRCs in the groups described in (D). Data are presented as mean ± SEM from 3 or more independent

experiments. Statistical significance was determined using a one-way ANOVA multiple comparison test. *$P < 0.05$, **$P < 0.01$, ***$P < 0.001$, ****$P < 0.0001$; ns, not significant.
